# Computer simulation models as a tool to investigate the role of microRNAs in osteoarthritis

**DOI:** 10.1371/journal.pone.0187568

**Published:** 2017-11-02

**Authors:** Carole J. Proctor, Graham R. Smith

**Affiliations:** 1 Newcastle University Institute for Ageing, Newcastle University, Newcastle upon Tyne, United Kingdom; 2 Institute of Cellular Medicine, Newcastle University, Newcastle upon Tyne, United Kingdom; 3 Bioinformatics Support Unit, Faculty of Medical Sciences, Newcastle University, Newcastle Upon Tyne, United Kingdom; University of Massachusetts Medical School, UNITED STATES

## Abstract

The aim of this study was to show how computational models can be used to increase our understanding of the role of microRNAs in osteoarthritis (OA) using miR-140 as an example. Bioinformatics analysis and experimental results from the literature were used to create and calibrate models of gene regulatory networks in OA involving miR-140 along with key regulators such as NF-κB, SMAD3, and RUNX2. The individual models were created with the modelling standard, Systems Biology Markup Language, and integrated to examine the overall effect of miR-140 on cartilage homeostasis. Down-regulation of miR-140 may have either detrimental or protective effects for cartilage, indicating that the role of miR-140 is complex. Studies of individual networks in isolation may therefore lead to different conclusions. This indicated the need to combine the five chosen individual networks involving miR-140 into an integrated model. This model suggests that the overall effect of miR-140 is to change the response to an IL-1 stimulus from a prolonged increase in matrix degrading enzymes to a pulse-like response so that cartilage degradation is temporary. Our current model can easily be modified and extended as more experimental data become available about the role of miR-140 in OA. In addition, networks of other microRNAs that are important in OA could be incorporated. A fully integrated model could not only aid our understanding of the mechanisms of microRNAs in ageing cartilage but could also provide a useful tool to investigate the effect of potential interventions to prevent cartilage loss.

## Introduction

MicroRNAs (miRNAs) are involved in many signalling pathways, especially those involving gene regulation [1]. They are known to enhance degradation of mRNAs and also to inhibit translation of mRNAs by a variety of mechanisms. It is known that miRNAs play an important role in complex networks that are enriched with positive and negative feedback loops. This has led to many researchers taking a systems biology approach whereby experimental data is integrated with mathematical modelling, e.g., review by Vera et al, 2013 [2]. Previous models have used functions to represent inhibition and activation in a series of ordinary differential equations using deterministic algorithms. These models have provided useful insight into the expected behaviour of the system due to the presence of miRNAs in different network motifs (small regulatory circuits that recur in gene networks) [3], such as positive feedback, negative feedback, and feedforward loops. In this study, we considered these motifs but unlike previous models, we explicitly modelled the interactions between different components using mass action kinetics. Since many molecular numbers such as genes and mRNA may be low, we also used stochastic simulation.

Positive feedback loops occur where two components in a system either positively or negatively regulate each other. Whereas positive regulation leads to signal amplification and a longer lasting cellular response, negative regulation may lead to bistability with one of the components staying switched on whilst the other is turned off. Since miRNAs are mainly involved in negative regulation, we only considered the latter situation. For example, a transcription factor (TF) may inhibit the synthesis of a miRNA that, in turn, enhances the degradation of the TF mRNA (Figures A-B in S1 File). Negative feedback occurs when two components in a system regulate each other in an incoherent fashion, e.g., component A activates component B whereas B inhibits A (Figure C in S1 File). This provides a homeostatic mechanism and is a very common motif in protein signalling and transcriptional networks leading to oscillatory behaviour ([4,5]) (Figure D in S1 File).

A coherent feedforward loop may either consist of a double positive or a double negative interaction (Figure E in S1 File illustrates a double negative loop). They function as sign-sensitive delay elements, extend the duration of target repression, or may prevent leaking of target genes. We only considered double negative loops in this study. In this type of loop the miRNA mediates a delay in the response of target gene to TF (see Vera *et al*. Fig 4.6) [2]. On the other hand, incoherent feedforward loops produce accelerated and pulse-like responses to signals (Figure F in S1 File). It has been suggested that the function of miRNAs in these loops is to provide fine-tuning and that they act as noise buffers [6].

MiRNAs have been implicated in many diseases such as cancer [7], neurodegeneration [8], and osteoarthritis (OA) [9]. OA is a disease of the joints characterised by loss of cartilage, abnormal bone growth, e.g., osteophytes, and changes to the synovium. Once thought to be mainly a degenerative disorder, it is now considered that inflammation may be important in disease progression. The cartilage mainly consists of an extracellular matrix (ECM) with chondrocytes (the only cell type) sparsely distributed throughout the ECM. Chondrocytes maintain the cartilage by responding to signals (growth factors and cytokines) to up-regulate anabolic and catabolic processes. There is evidence from several studies that miRNAs are involved in regulating gene expression in signalling pathways that control cartilage turnover [10–14]. In particular, miR-140 has been consistently found to have a significant role in cartilage ageing and the development of OA in studies from different groups [11–13,15–17]. We confirmed its importance (and that of other miRNAs such as miR-455 [18,19]) by integrating information on miRNA targets and OA-associated genes. In view of this, we started by investigating the mechanisms of gene regulation by miR-140 using experimental data from the literature and the above bioinformatics result. We also used the bioinformatics results to search for potential novel miRNAs for OA.

To date, computational models of the role of miRNAs in OA are lacking, although models have been developed in oncogenesis and ischemic vascular disease [20–22]. Therefore, we adapted the generic models (described in S1 File) and constructed five different networks based on experimental evidence from the literature, where miR-140 is involved in signalling pathways relevant to OA [10–13,17]. We used the dynamical behaviour of the relevant components in the network to infer the type of motif connecting miR-140 to the other components. Finally we integrated the five individual models to examine the overall effect of miR-140 on cartilage turnover. The models demonstrated how computational modelling can help to increase our understanding of the role of miRNAs in OA and highlighted the need for integrated approaches.

## Methods

### Modelling

The models were constructed in the Systems Biology Markup Language (SBML) [23] using the Python tool SBML shorthand [24]. SBML is a modelling standard that allows for model sharing and development and enables models to be simulated in a wide range of freely available software tools. The models were simulated using COPASI 4.18 [25] and tools developed at Newcastle University [26] (further details below). The network diagrams were constructed in CellDesigner [27], which uses the Systems Biology Graphical Notation [28]. The model output was analysed in R and plotted with ggplot2 [29].

### Stochastic simulation

We mainly used stochastic simulation, which is based on the Gillespie algorithm (direct method) [30]. This algorithm simulates every reaction, updating the number of molecules of each species after a reaction occurs. At each time-step random numbers are generated to determine the next reaction to occur and the time interval to that reaction. The probability of any particular reaction occurring is proportional to its associated parameter value and the number of substrate molecules. Each stochastic simulation produces a different trajectory. Therefore, multiple stochastic simulations were carried out on a cluster using software based on the Gillespie algorithm that was developed at Newcastle University [26]. The input for the simulation was the duration time (virtual time) in seconds (typically the equivalent of 4 or 8 hours), the number of intervals to be saved to an output file (1000), and the initial conditions and parameter values (which are contained in the SBML files and can also be found in S1 File).

### Deterministic simulation

For deterministic simulations, we used the LSODA algorithm in COPASI, a method for solving stiff and non-stiff systems of ordinary differential equations by automatic selection [31]. We used the COPASI default parameters for this solver (relative tolerance = 1e-6; absolute tolerance = 1e-13; maximum internal step size = 10000).

### Kinetic parameters

It is rarely possible to obtain all the kinetic parameters required for a mathematical model due to a lack of suitable time-course experimental data. Even when data are available, they may not be appropriate as parameter values are usually dependent on cell type and experimental conditions. Therefore, we initially chose parameters with an order of magnitude that would be expected for the process, e.g., degradation rates were chosen to give half-lives that were in an appropriate range), and then synthesis rates were set to obtain the chosen steady state level. In the model of miR-140 in the TGF-β signalling pathway, parameters were chosen to fit experimental data that shows that Smad3 is rapidly phosphorylated after the addition of TGF-β (peaking at 30–45 minutes), and due to negative feedback is de-phosphorylated by 8 hours, although still above basal at this time-point [32].

### Availability of models

The models were deposited in Biomodels [33] and assigned the identifiers [MODEL1610100000-1610100004, MODEL1705170000-1705170005]. Full details of all the model components, reactions, and parameters are given in S1 File.

#### Bioinformatics analysis

**Fig 1:**
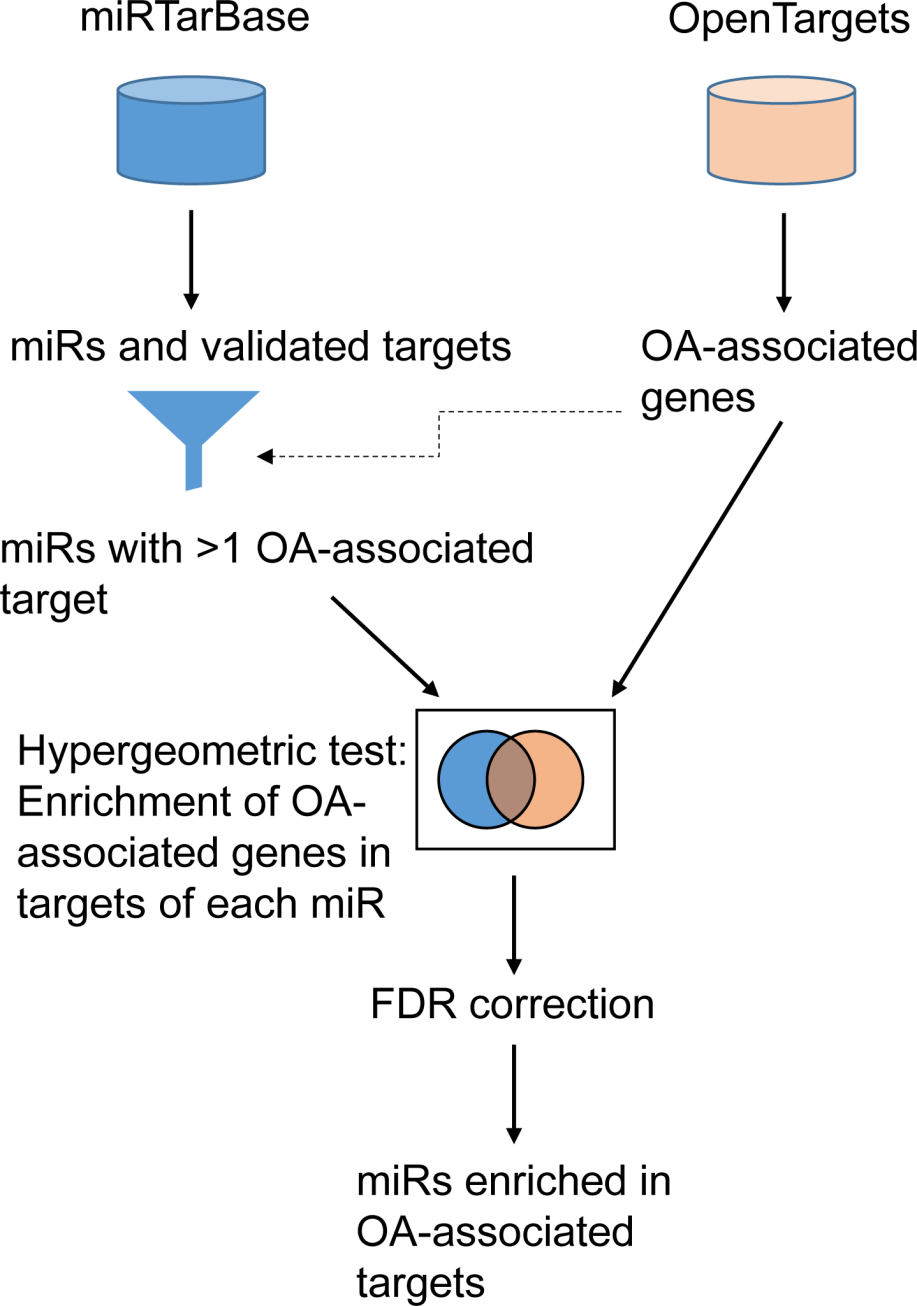
Identification and prioritisation of possible OA-associated miRNAs. Schematic representing the integration of information about (left) validated targets of miRNAs and (right) OA-associated genes. The output is a list of miRNAs sorted by the multiple-test-corrected p-value of their enrichment in OA-associated targets.

Validated targets of all human miRNAs were downloaded from miRTarBase [34] and genes annotated as associated with OA from Open Targets (www.targetvalidation.org) [35]. The miRNAs were filtered to retain only those whose targets include more than one OA-associated genes (2125 miRNAs), and the hypergeometric test was used to assess the enrichment of the OA-related targets among the entirety of the targets of each miRNA (Fig 1). The details of the searches are given below.

### miRTarBase

http://mirtarbase.mbc.nctu.edu.tw/php/download.php. hsa_MTI.xlsx (Human data), release 6.1, was downloaded Feb 20 2017 and converted from xlsx format to text csv with libreoffice. All interactions were included whether evidence was annotated as strong or weak.

### Open targets

https://www.targetvalidation.org/disease/EFO_0002506/associations. The search term for target or disease was "osteoarthritis". The results were downloaded on Feb 20 2017, at which time there were 1202 targets. All types of evidence of association in the database for this disease were included (genetic associations (18), drugs (120), text mining (1k) and animal models (32)).

Data from miRTarBase and Open Targets were integrated with an R script (S1 Script).

### Search for novel miRNAs

In addition to miR-140-5p, a miRNA that is well-known to have a role in OA, we used the bioinformatics analysis to find novel miRNAs that may have a potential role in OA. We looked for those miRNAS that had targets with strong validation evidence and that had not been previously examined in the context of OA. We first carried out a search in PubMed (https://www.ncbi.nlm.nih.gov/pubmed/, date of search: July 11 2017), using the terms “(miR-Xy-Zp OR miR-Xy) AND osteoarthritis” (e.g. (miR-200c-3p OR miR-200) AND osteoarthritis) to establish whether the chosen miRNA was novel. We included the generic miRNA term in the search due to many publications not specifying exactly which miRNA was being studied. For the potential novel miRNAs, a further search was carried out in PubMed (date of search: July 17 2017) on the targets with strong validation evidence using the terms (GeneX AND (cartilage OR osteoarthritis)), where GeneX is the HUGO approved symbol of the target gene (http://www.genenames.org/).

## Results

### Bioinformatics analysis confirm the importance of miR-140 in OA

We started by integrating data on validated miRNA targets and OA-related genes to identify those miRNAs having an unusually high proportion of OA-related targets. When sorted by hypergeometric p-value, miR-140 ranks 12th, with a multiple test-corrected p-value of 0.049 and an enrichment (observed to expected number of OA-related targets) of 3.7. This is the highest of any miRNA that has 10 or more OA-related targets, suggesting that its function may be particularly biased towards regulation of cartilage. This data is shown in S2 File.

### Models of regulatory feedback loops demonstrate how miRNAs affect gene regulation

We constructed a set of generic models to illustrate the behaviour of miRNAs in four different feedback loops, namely positive feedback, negative feedback, coherent feedforward, and incoherent feedforward (Figures A-F in S1 File). Full details of these models and the results are given in S1 File.

### miR-140 is involved in network motifs relevant to OA

We chose five targets of miR-140 that have been studied in the context of OA. Three of these were validated OA-related targets that were in the list from miRTarBase, namely *MMP13*, *HDAC4* and *IGFBP5* (S2 File). The other two were *ADAMTS5* and *SMAD3*. *ADAMTS5* is a predicted target and has been validated in the literature [36]. According to the microRNA databases, *SMAD3* is not a predicted or a validated target but there is evidence that SMAD3 is a direct target of miR-140 at the protein level [12]. Since TGF-β signalling is important in maintaining cartilage homeostasis, we considered that the SMAD3/miR-140 interaction was worthy of further investigation. The quality of the chosen studies are variable and the authors would like to point out that we do not claim that the all the experimental results on which we have based our models is accurate.

### A model of miR-140 in TGF-β signalling–a positive feedback loop

**Fig 2:**
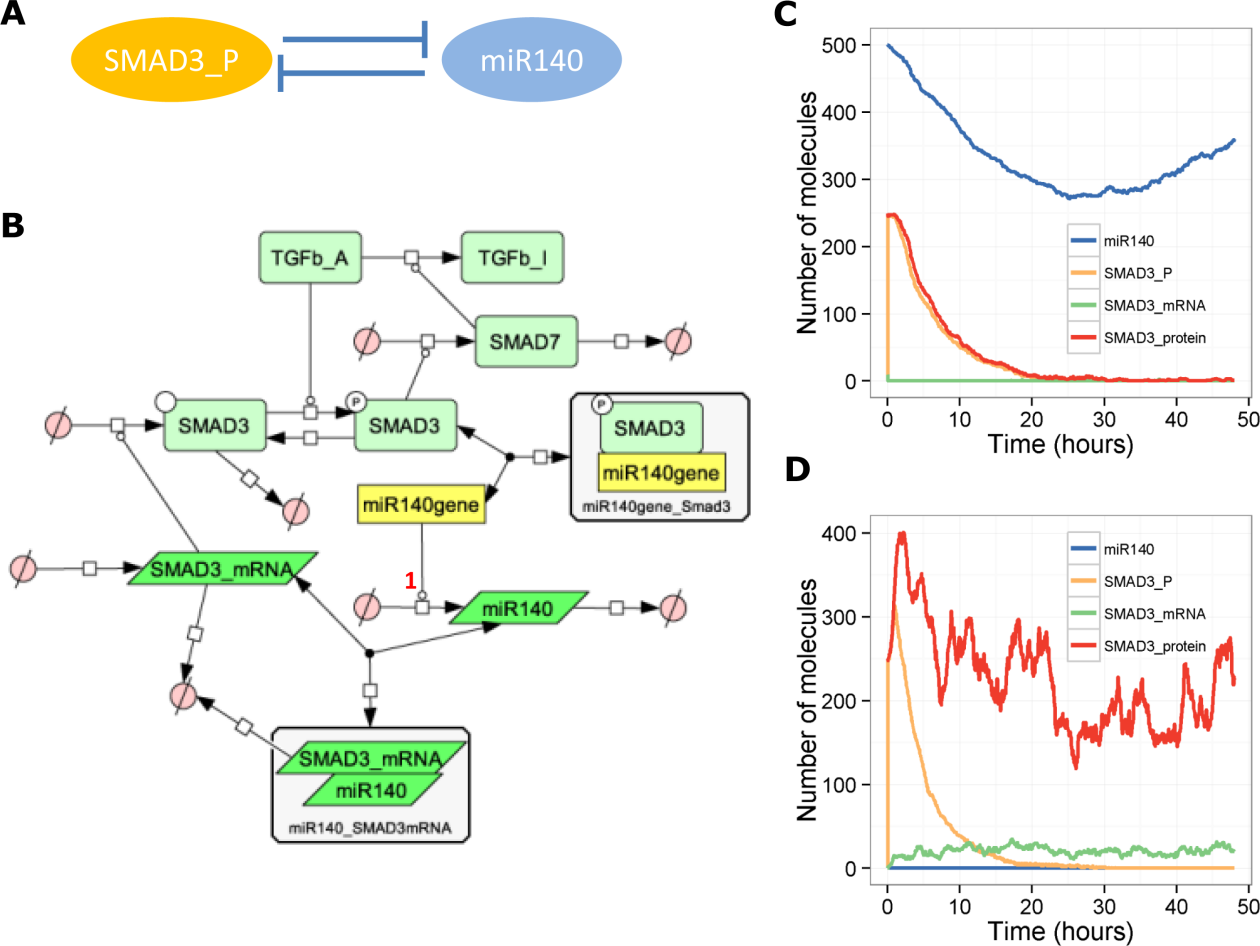
Model of the involvement of miR-140 in SMAD3 signalling. **A**, Network motif showing positive feedback. **B**, Diagram of full model. **C**-**D,** Output from one stochastic simulation with miR-140 present (miR140 = 500 initially, *k*_*synmiR140*_ = 0.0018 s^-1^) (C) or without miR-140 (miR140 = 0 initially, *k*_*synmiR140*_ = 0.0 s^-1^) **D**. Key for **B**: TGFb_A–active TGF-β, TGFb_I–inactive TGF-β, miR140_SMAD3mRNA–SMAD3 mRNA bound by miR140 to inhibit its translation.

It is known that the growth factor TGF-β usually has a protective role in cartilage as it normally up-regulates anabolic genes via the ALK5/SMAD2/3/4 pathway. A study by Pais *et al*. showed that TGF-β signalling promotes phosphorylation of SMAD3, which in turn binds to the promoter of the miR-140 gene to inhibit transcription [12]. In addition miR-140 inhibits production of SMAD3 protein but does not affect levels of *SMAD3* (mRNA) [12]. This data suggests that miR-140 binds to *SMAD3* mRNA to inhibit its translation rather than enhancing its degradation. This is an example of a positive feedback loop mediated by a double negative loop as shown in Fig 2A. We constructed a simple model of this system (Fig 2B). Pais *et al*. show that miR-140 is transiently suppressed by TGF-β and that levels return to basal by 48 hours [12]. This is due to negative feedback loops in signalling pathways, which act to ensure that signalling is only transient to prevent adverse effects from over-activation. A well-known negative feedback loop in TGF-β signalling is the up-regulation of the inhibitory SMAD7, which may enhance degradation of TGF-β receptors and prevent phosphorylation of SMAD3. Therefore, we included SMAD7 in the model, and, for simplicity, we assumed that SMAD7 directly inactivated TGF-β. To model this we included two pools of TGF-β, either active (TGFb_A) or inactive (TGFb_I). We assumed that TGF-β is initially activated which leads to rapid phosphorylation of SMAD3 (Fig 2C and 2D). If miR-140 levels are initially high, then after SMAD3 phosphorylation, miR-140 declines but then levels recover again due to the negative feedback via SMAD7, which inactivates TGF-β. However, miR-140 is always sufficient to prevent an increase in *SMAD3* (Fig 2C). If we assume miR-140 is absent, then after activation of TGF-β, there is an increase in *SMAD3* transcription and translation and therefore a higher peak in phospho-SMAD3 although the duration of the signalling response is not affected (Fig 2D). This suggests that miR-140 only acts to dampen the initial response.

### A model of miR-140 in the SOX9 pathway–an incoherent feedforward loop

**Fig 3:**
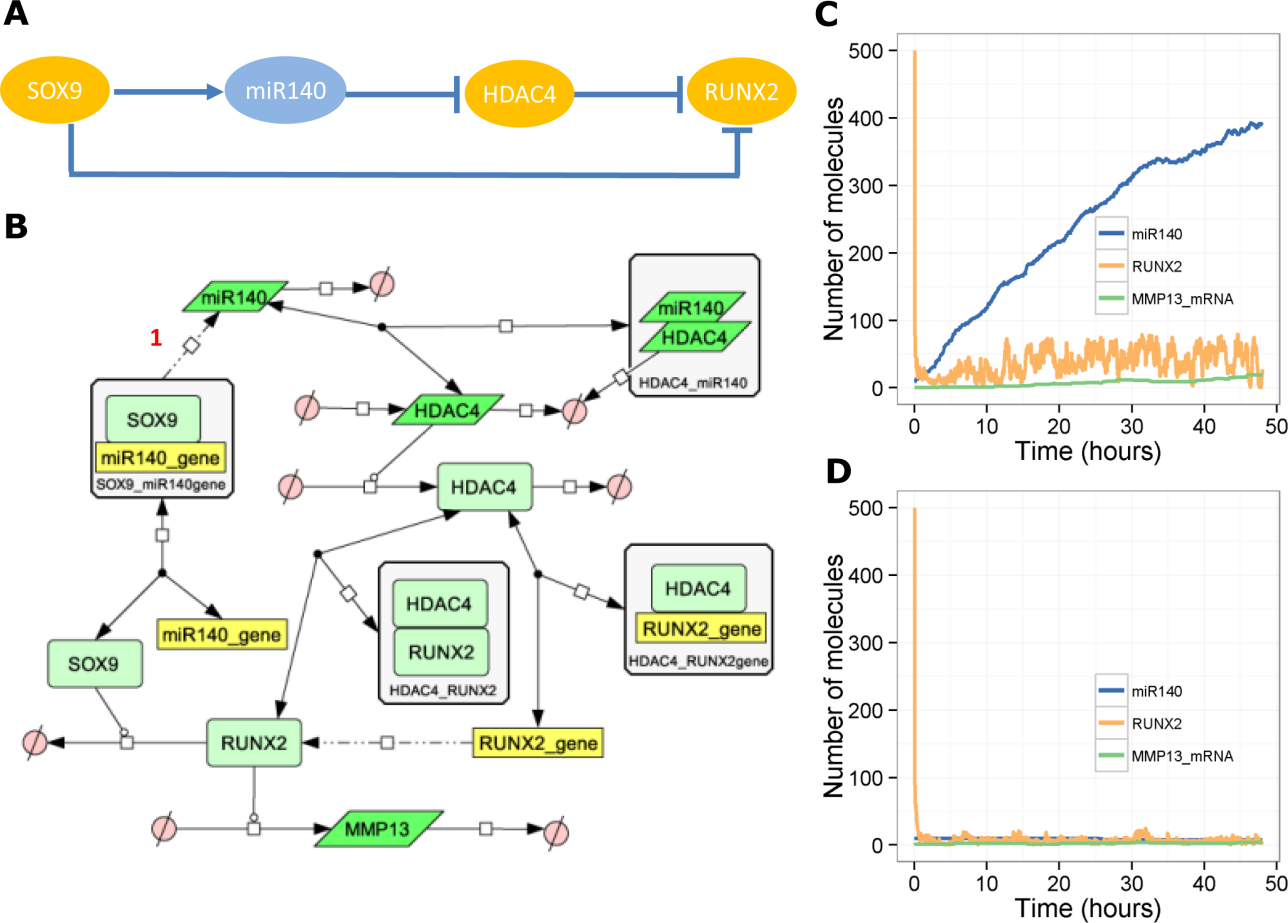
Model of the involvement of miR-140 in SOX9-dependent regulation of RUNX2. **A**, Network motif showing incoherent feedforward loop. **B**, Diagram of full model. **C**-**D**, Output from one stochastic simulation, **C,** with miR-140 (*k*_*synmiR140*_ = 0.0018 s^-1^) or **D**, without miR-140 (*k*_*synmiR140*_ = 0.0 s^-1^).

SOX9 is a transcription factor that is important in chondrogenesis and cartilage development. It inhibits RUNX2, a transcription factor that inhibits chondrocyte proliferation, up-regulates the matrix-degrading enzyme, *MMP13*, and promotes chondrocyte hypertrophy [37]. SOX9 inhibits RUNX2 by promoting its degradation [38]. HDAC4 also inhibits RUNX2 by binding to its promoter and inhibiting transcription. In addition, HDAC4 binds to RUNX2 protein to inhibit its transcriptional activity. A target of SOX9 is miR-140 [18,39], which has been shown to inhibit HDAC4 [13]. Since HDAC4 inhibits RUNX2, the overall effect is that miR-140 activates RUNX2. Therefore SOX9 directly inhibits RUNX2 but also indirectly activates RUNX2 via miR-140 (Fig 3A). This is an example of an incoherent feedforward loop, but note that this has different features to the motif described above. In the generic model, a TF directly activated a target but indirectly inhibited the target via a miRNA. In this example, a TF (SOX9) directly inhibits a target (RUNX2) and indirectly activates it via a miRNA (miR-140). Therefore, we would not expect a pulse-like response but would expect miR-140 to have a moderating effect on RUNX2 inhibition. We constructed a simple model, where we assumed that SOX9 up-regulates miR-140, miR-140 binds to *HDAC4* mRNA to inhibit its translation, HDAC4 protein binds to RUNX2 to inhibit its activity and also binds to the *RUNX2* gene to inhibit transcription, and SOX9 promotes the degradation of RUNX2 protein (Fig 3B). Hence SOX9 directly inhibits RUNX2 but also has a delayed response whereby it activates RUNX2. Therefore, we would predict that on activation of SOX9, RUNX2 levels would decline, but after up-regulation of miR-140, pools of RUNX2 would stabilise. The model output shows this behaviour as RUNX2 levels decline in the presence of SOX9, but the increase in miR-140 leads to a slight recovery in RUNX2 levels and so *MMP13* levels increase slightly (Fig 3C). In the absence of miR-140, RUNX2 declines to very low levels and so no *MMP13* is produced (Fig 3D). Therefore miR-140 moderates the response of RUNX2 to SOX9, raising the steady-state level of RUNX2.

### A model of miR-140 in the IL-1 pathway–a coherent feedforward loop

**Fig 4:**
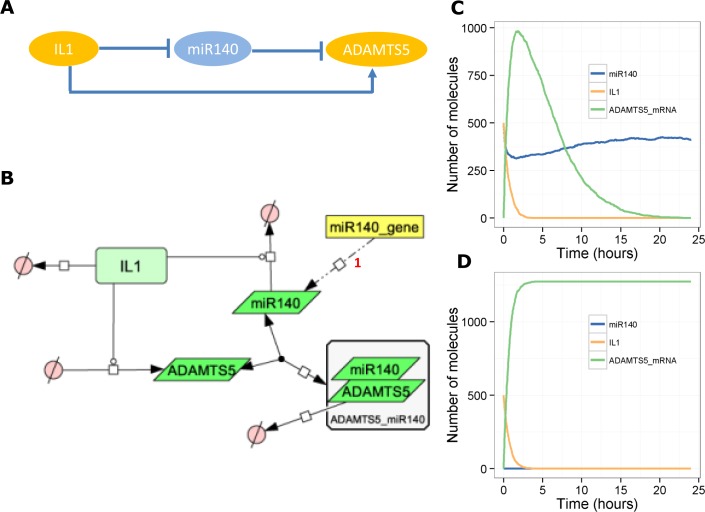
Model of the interaction between miR-140, IL-1 and *ADAMTS5*. **A**, Network motif showing coherent feedforward loop. **B**, Diagram of full model. **C**-**D**, Output from one stochastic simulation, **C,** with miR-140 (miR140 = 500 initially, *k*_*synmiR140*_ = 0.0018 s^-1^) or **D,** without miR-140 (miR140 = 0 initially, *k*_*synmiR140*_ = 0.0 s^-1^).

The cytokine IL-1 activates signalling pathways that lead to up-regulation of catabolic genes such as *MMP1*, *MMP13* and *ADAMTS5* [40]. It has been shown that chondrocytes treated with IL-1β have reduced expression of miR-140 [11]. In the same study, treating cells with ds-miR-140 (a mimic of miR-140) down-regulates IL-1β-induced expression of *ADAMTS5* [11]. In light of this data, we constructed a simple model in which IL-1 leads to up-regulation of *ADAMTS5*, which is then degraded by miR-140. In addition, we assumed that IL-1 increases the rate of miR-140 degradation as observed [11], resulting in a coherent feedforward loop (Fig 4A and 4B). The presence of miR-140 provides a pulse-like response in the up-regulation of *ADAMTS5* (Fig 4C), whereas in the absence of miR-140, the model output shows *ADAMTS5* continuing to increase even after a decline in levels of IL-1 (Fig 4D).

### Model of the miR-140/IL1/*MMP13* pathway–an incoherent feedforward loop

**Fig 5:**
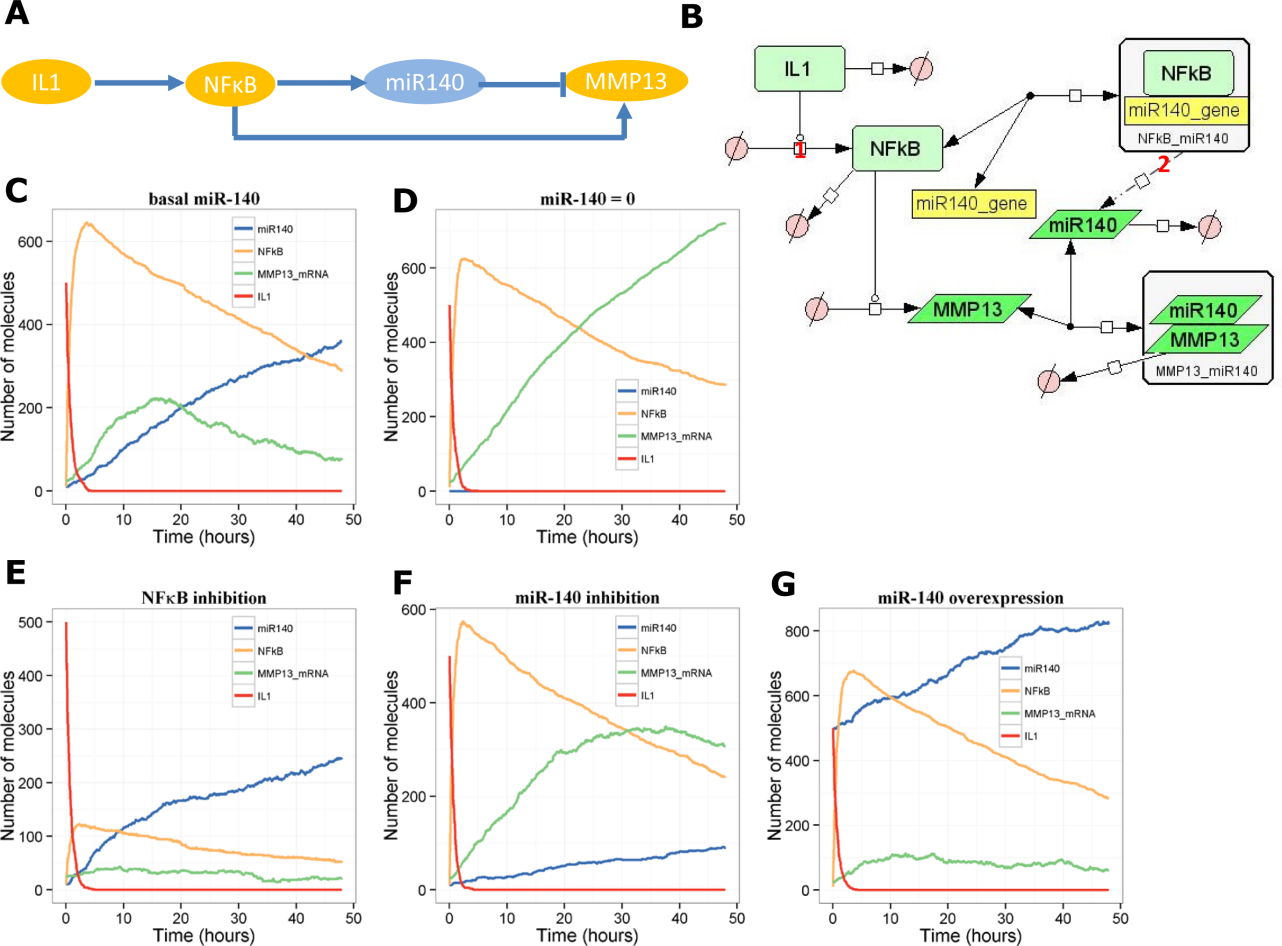
Model of the interaction between miR-140, IL-1 and *MMP13*. **A**, Network motif showing incoherent feedforward loop. **B**, Diagram of full model. **C**-**D**, Output from one stochastic simulation, **C**, with miR-140 or **D**, without miR-140. **E**-**G**, output showing simulated interventions: **E**, NFκB 80% inhibition (*k*_*actNFkB*_ = 1e-4 s^-1^); **F**, miR-140 50% inhibition (*k*_*synmiR140*_ = 0.0009 s^-1^); **G**, miR-140 overexpression (miR140 = 500 initially, *k*_*synmiR140*_ = 0.0036 s^-1^).

In contrast to Miyaki *et al*. [11], a study by Liang *et al*. showed that IL-1β up-regulates miR-140 in an NF-κB-dependent pathway in C28/C12 human cartilage cells [10]. They also showed that stimulating the cells with exogenous miR-140 reduced the levels of *MMP13* [10]. This is an example of an incoherent feedforward loop, and so we would expect a pulse-like response of *MMP13 (*the target) in the presence of miR-140. We constructed a network model (Fig 5A and 5B). Simulation output from the model shows that after a stimulus of IL-1, in the presence of miR-140, *MMP13* increases, peaking at about 10–12 hours and then declines (Fig 5C). When miR-140 is absent, *MMP13* keeps increasing over 48 hours even though the IL-1 signal is turned off after 2–3 hours (Fig 5D). We also used this model to examine the effect of simulated interventions such as NF-κB inhibition, miR-140 inhibition and miR-140 over-expression. NF-κB inhibition (by 80%) led to reduced levels of miR-140 and *MMP13* (Fig 5E). miR-140 inhibition (by 50%) led to an increase in *MMP13* (Fig 5F) and lastly, over-expressing miR-140 (two-fold increase) led to reduced levels of *MMP13* (Fig 5G).

### Model of miR140 in the IGF-1 signalling pathway–an incoherent feedforward loop

**Fig 6:**
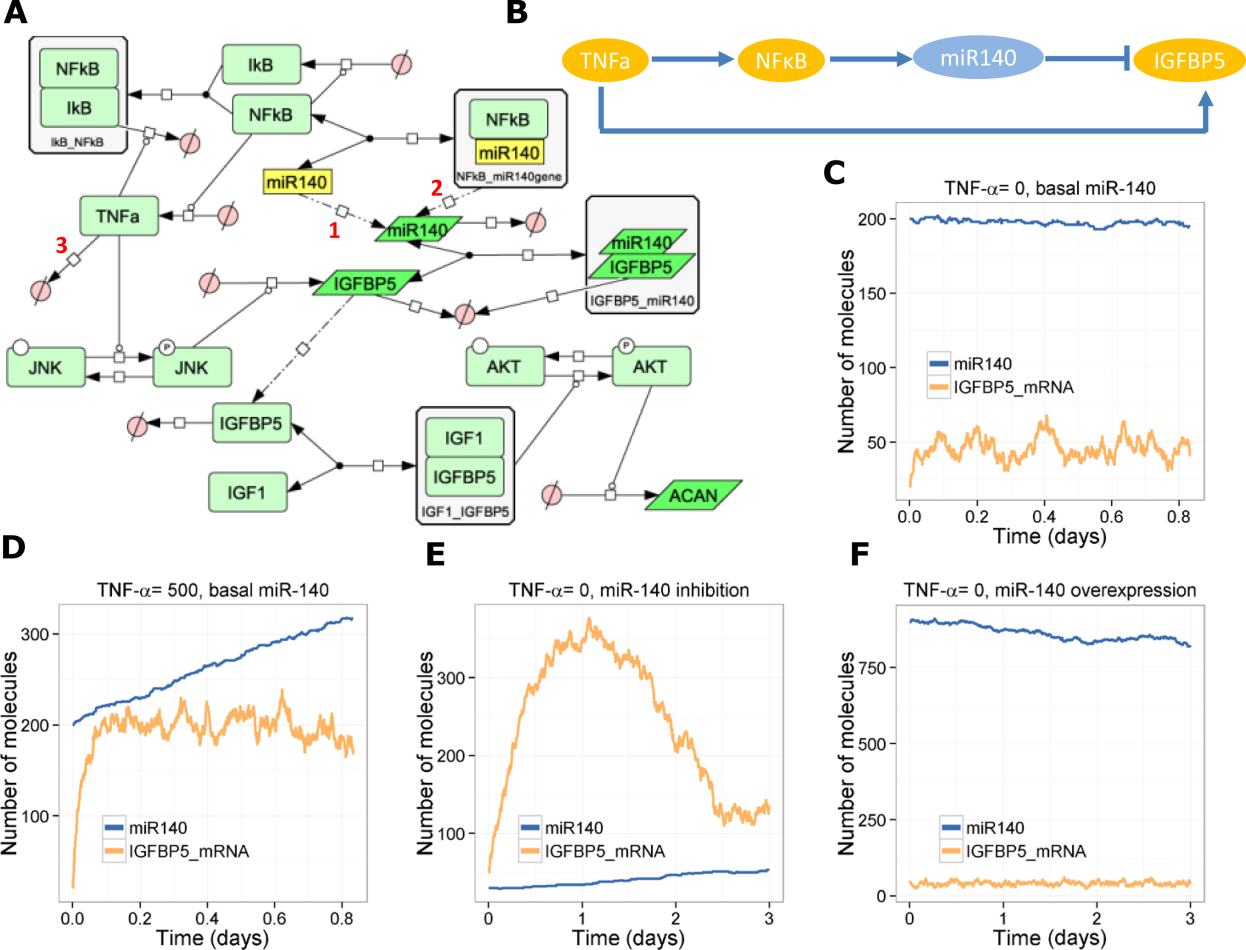
Model of the interaction between miR-140, IGFBP-5 and TNF-α. **A,** Diagram of full model; **B**, Network motif showing incoherent feedforward loop. **C**-**F**, Output from one stochastic simulation showing total pools of miR-140 and *IGFBP5* mRNA, **C**,TNF-α = 0; **D**, TNF-α = 500; **E**, TNF-α = 0, miR-140 inhibition (miR140 = 30 initially, *k*_*synmiR140*_ = 6e-5 s^-1^, *k*_*synmiR140NFkB*_ = 1.5e-4 s^-1^); **F**, TNF-α = 0, miR-140 overexpression (miR140 = 900 initially, *k*_*synmiR140*_ s^-1^ = 0.0018, *k*_*synmiR140NFkB*_ = 0.0045 s^-1^).

**Fig 7:**
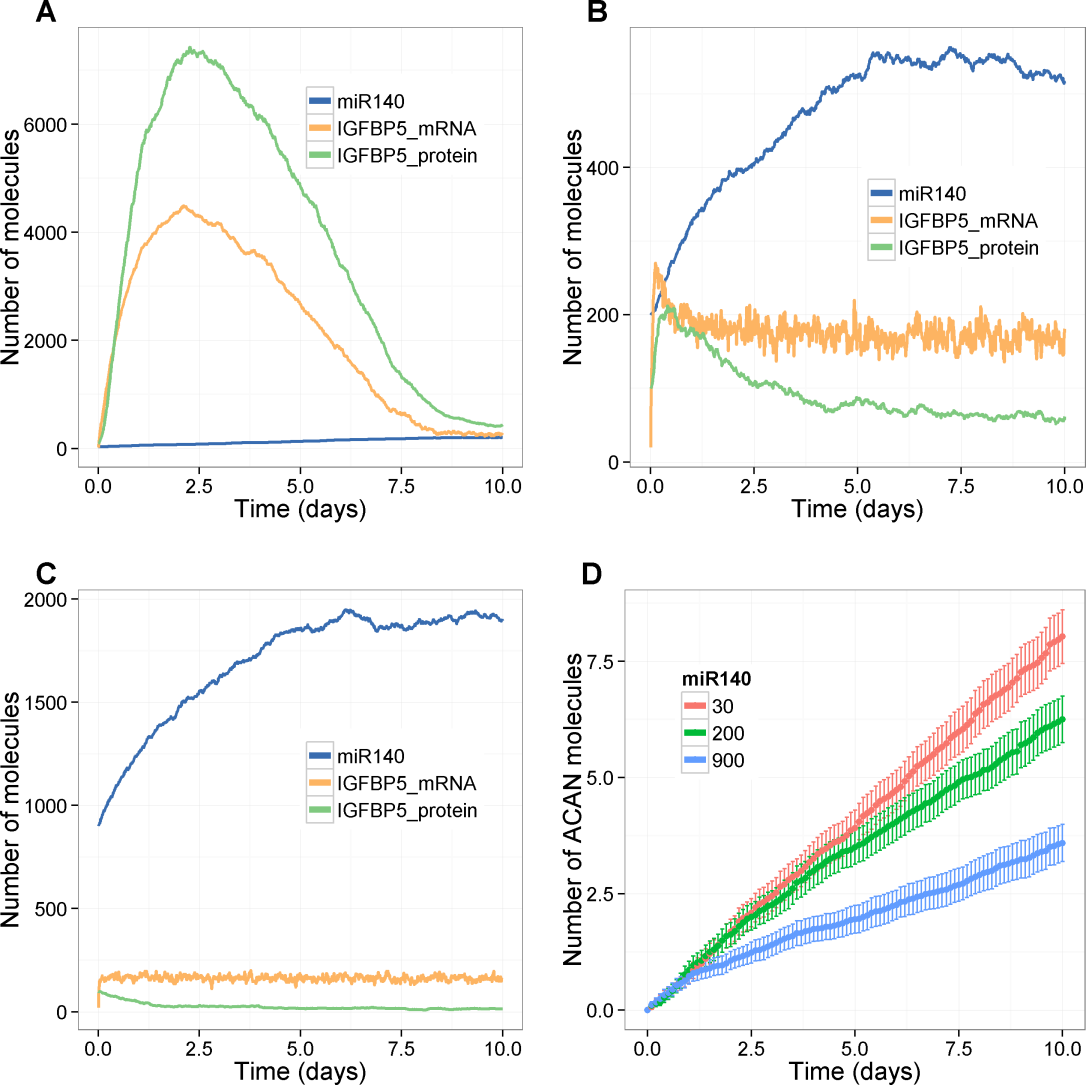
Effect of chronic activation of TNF-α on miR-140, Igfbp5 and Acan. **A**-**F**, Tnfa = 500 initially, *k*_*degTnfa*_ = 1e-5 **A**, Low levels of miR-140 (miR140 = 30 initially, *k*_*synmiR140*_ = 6e-5 s^-1^, *k*_*synmiR140NFkB*_ = 1.5e-4 s^-1^); **B**, Basal levels of miR-14-0 (mir140 = 200 initially, *k*_*synmiR140*_ = 4e-4 s^-1^, *k*_*synmiR140NFkB*_ = 1e-3 s^-1^; **C**, High levels of miR-140 (miR140 = 900 initially, *k*_*synmiR140*_ = 1.8e-3 s^-1^, *k*_*synmiR140NFkB*_ = 4.5e-3 s^-1^); **D**, Effect of miR-140 on *ACAN* levels (mean of 100 stochastic simulations; error bars indicate confidence interval of the mean).

IGF-1 signalling in chondrocytes leads to induction of anabolic genes and decreases the response to catabolic factors [41,42]. It has also been shown that IGF-1 signalling maintains chondrocytes in a proliferative state to prevent progression to hypertrophy [43]. The insulin-like growth factor binding protein-5 (IGFBP-5) is important for maintaining pools of IGF-1 in the joint [44]. *IGFBP5* is expressed in chondrocytes and has been shown to be a direct target of miR-140 [17]. *IGFBP5* mRNA is significantly reduced in OA compared to normal human chondrocytes and is significantly up-regulated in OA chondrocytes by the cytokines TNF-α, IFN-γ and IL-10 and by the growth factors TGF-β [17]. We constructed a model describing the interactions between TNF-α, NF-κB, miR-140, and *IGFBP5* (Fig 6A), which we show to be an incoherent feedforward loop (Fig 6B). We also included JNK and AKT as mediators in the pathway and *ACAN* as readout of IGF-1 signalling (Fig 6A). We calibrated our model based on data from Tardif *et al*. [17] so that the addition of TNF-α led to approximately a three-fold increase in *IGFBP5* mRNA and a 1.5-fold increase in miR-140 at 20 hours (Fig 6C and 6D). The model was simulated to check that miR-140 inhibition and over-expression resulted in an increase or decrease of *IGFBP5* mRNA respectively (Fig 6E and 6F), as shown experimentally [17]. The model was then used to examine the effects of chronic activation of TNF-α with low, medium (basal), or high levels of miR-140. If miR-140 is low initially, IGFBP-5 mRNA and protein levels increase and peak at about 2.5 days, but then decline due to the increase in NF-κB activity and a gradual increase of miR-140 to basal levels (Fig 7A). If miR-140 levels are initially at basal levels, the model suggests that chronic activation of TNF-α leads to up-regulation of miR-140 due to activation of NF-κB, an increase in *IGFBP5* mRNA due to activation of JNK but a decline in IGFBP-5 protein due to targeting of *IGFBP5* mRNA for degradation by miR-140 (Fig 7B). Finally, if miR-140 is over-expressed when there is chronic TNF-α-activation, miR-140 levels increase further resulting in very low levels of IGFBP-5 protein despite the increase in *IGFBP5* transcription (Fig 7C). We also looked at the effect of chronic TNF-α-activation and miR-140 levels on production of Aggrecan (*ACAN*). The model predicted that increasing miR-140 results in less *ACAN* due to the inhibitory effect on IGFBP-5 translation and so less IGF-1 signalling (Fig 7D).

### A combined model of the effects of miR-140 in OA

**Fig 8:**
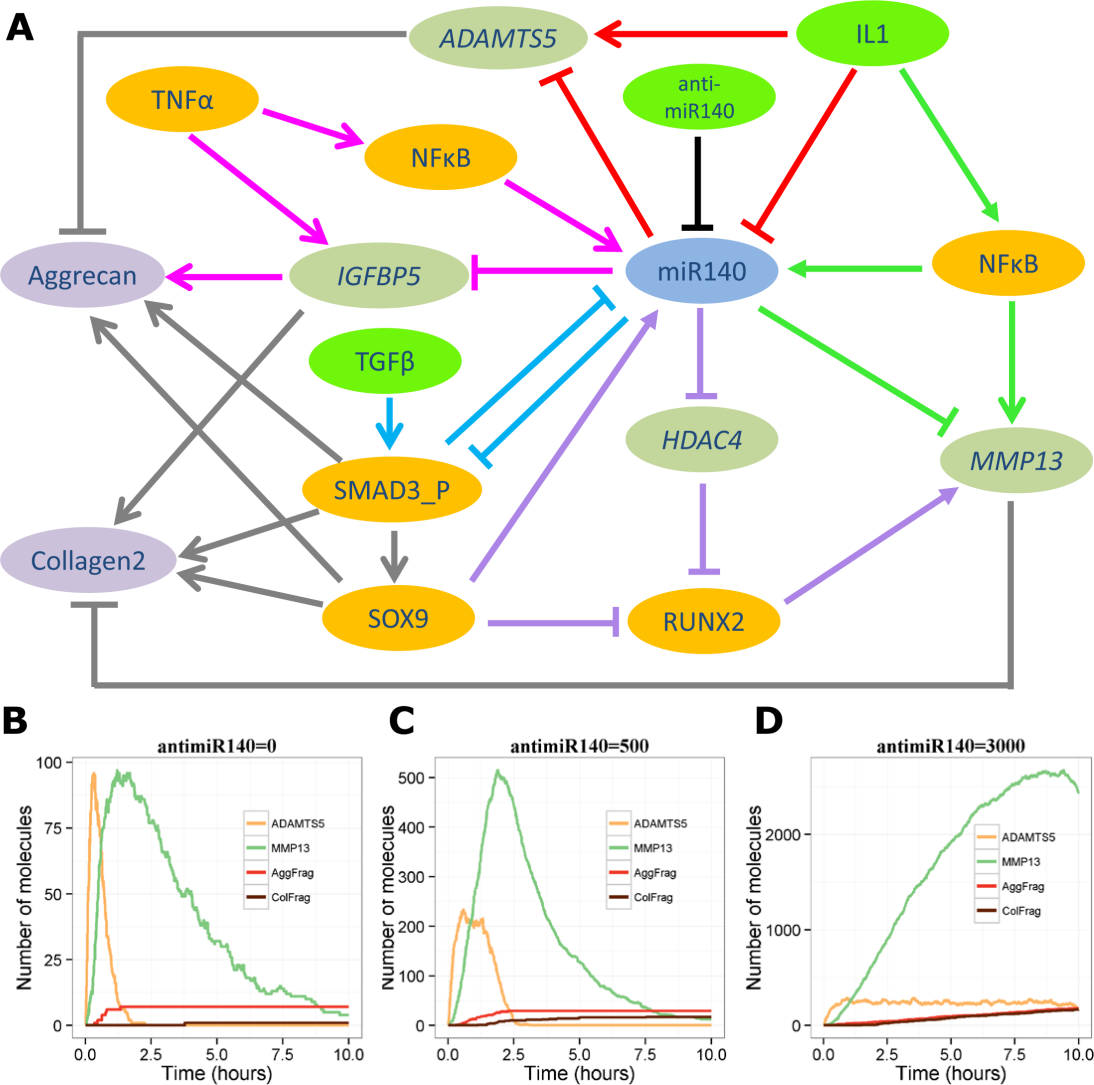
Integrated model:: Inhibition of miR-140. **A**, Diagram showing main components. Arrows indicate activation, blocked lines indicate inhibition. The red lines show coherent feedforward motif from miR-140/IL1/ADAMTS model; turquoise lines show postive feedback motif from the miR-140/TGF-β/SMAD3 model; green lines show the incoherent feedforward motif from the miR-140/IL1/MMP13 model; lilac lines show the incoherent feedforward motif from the miR-140/SOX9 model; pink lines show the incoherent feedforward motif from the miR-140/IGF-1/TNFα model; dark grey lines indicate additional reactions for the combined model. **B**-**D**, Output from the stochastic integrated model after a combined stimulus of IL-1, TNF-α and TGF-β showing levels of ADAMTS-5 and MMP-13 protein, and the amount of Aggrecan and Collagen2 degradation (AggFrag and ColFrag, respectively). B, no inhibition of miR-140 (anti-miR140- = 0). C, moderate inhibition of miR-140 (anti-miR140 = 500); D, high inhibition of miR-140 (anti-miR140 = 3000).

The five individual models of the role of miR-140 in OA were combined to form an integrated model. The main components showing the different motifs are shown in Fig 8A. We used ADAMTS-5 and MMP-13 protein, aggrecan fragments (AggFrag) and collagen2 fragments (ColFrag) as readouts. First we investigated the effect of a combined stimulation of IL-1, TNF-α and TGF-β at the start of the simulation with different levels of miR-140 inhibition (anti-miR140). The model output shows that with no miR-140 inhibition both ADAMTS-5 and MMP-13 peak but then decline as IL-1 is degraded and there is very little degradation of Aggrecan or Collagen2 (Fig 8B). With moderate inhibition of miR-140, ADAMTS-5 and MMP-13 have much higher levels in the first three days leading to degradaton of matrix components, but eventually the increased synthesis of miR-140 overwhelms the effect of the anti-miR140 (Fig 8C). If very high levels of anti-miR140 are added at the start of the simulation, then both ADAMTS-5 and MMP-13 peak to much higher levels and stay in the system for much longer leading to more degradation of matrix components (Fig 8D).

**Fig 9:**
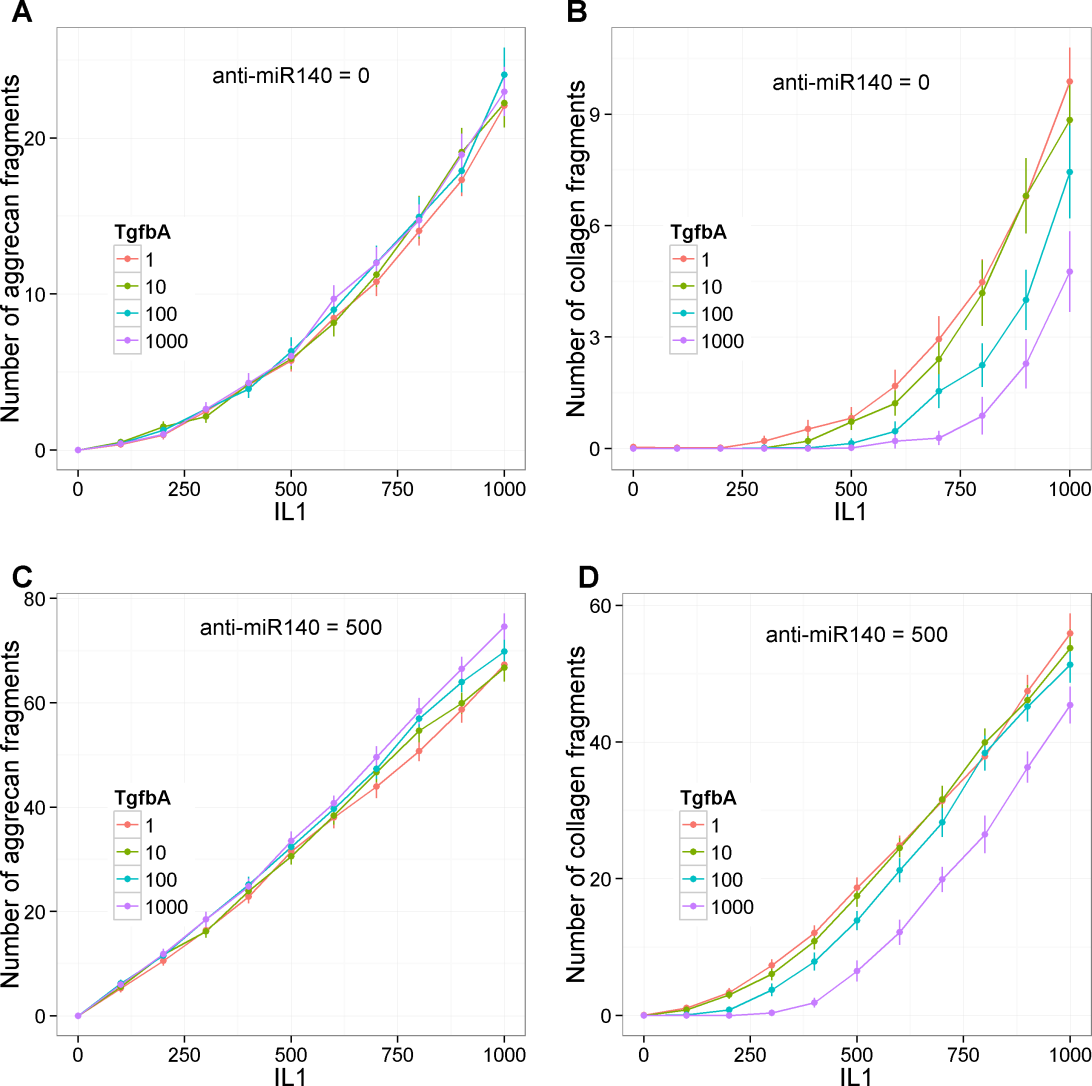
Integrated model: Combined effect of IL-1, TGF-β and miR-140 on cartilage degradation. Model output showing effect of varying the initial amounts of IL-1 (0–1000, 10 intervals) and TGF-β (1–1000, 3 intervals, log scale) simultaneously on levels of aggrecan fragments (**A**,**C**) or collagen2 fragments (**B**,**D**). **A**-**B**, no miR-140 inhibition (anti-miR140 = 0), **C**-**D**, miR-140 inhibition (anti-miR-140 = 500). Curves show mean of 100 stochastic simulations, error bars indicate 95% confidence interval of the mean.

To examine the interaction of the effects of miR-140, IL-1 (a catabolic factor) and TGF-β (normally, an anabolic factor) on cartilage degradation, the integrated model was then run with the initial levels of IL-1 and TGF-β being varied simultaneously with and without inhibition of miR-140. As levels of aggrecan and collagen2 fragments are low, the same combination of IL-1 and TGF-β can produce different levels of fragments in repeated simulations. Therefore, each combination of initial conditions was simulated 100 times and the mean and 95% confidence interval of the mean was plotted (Fig 9). The model output shows that both aggrecan and collagen2 fragments increase with increasing levels of IL-1 with much higher levels when miR-140 is inhibited. Increasing TGF-β has no effect on aggrecan degradation but significantly decreases collagen degradation when miR-140 is not inhibited (Fig 9A and 9B). However, when miR-140 is inhibited, increasing TGF-β leads to a slight increase in aggrecan fragments, when IL-1 > 700, but a significant decrease in collagen fragments and also delays the threshold level of IL-1 at which collagen fragments appear (Fig 9C and 9D). Interestingly, the model indicates that miR-140 may have an even greater effect than TGF-β on the threshold level of IL-1 necessary to initiate collagen degradation. Therefore the combined model suggests that overall miR-140 has protective effects on cartilage.

### Potential novel miRNAs in OA

Out of the top 12 miRNAs (S2 File), we found three miRNAs which to date had not been studied in the context of OA (zero publications in PubMed): miR-200c-3p, miR-100-5p and miR-1826 (S1 Table).

#### miR-200c-3p

**Fig 10:**
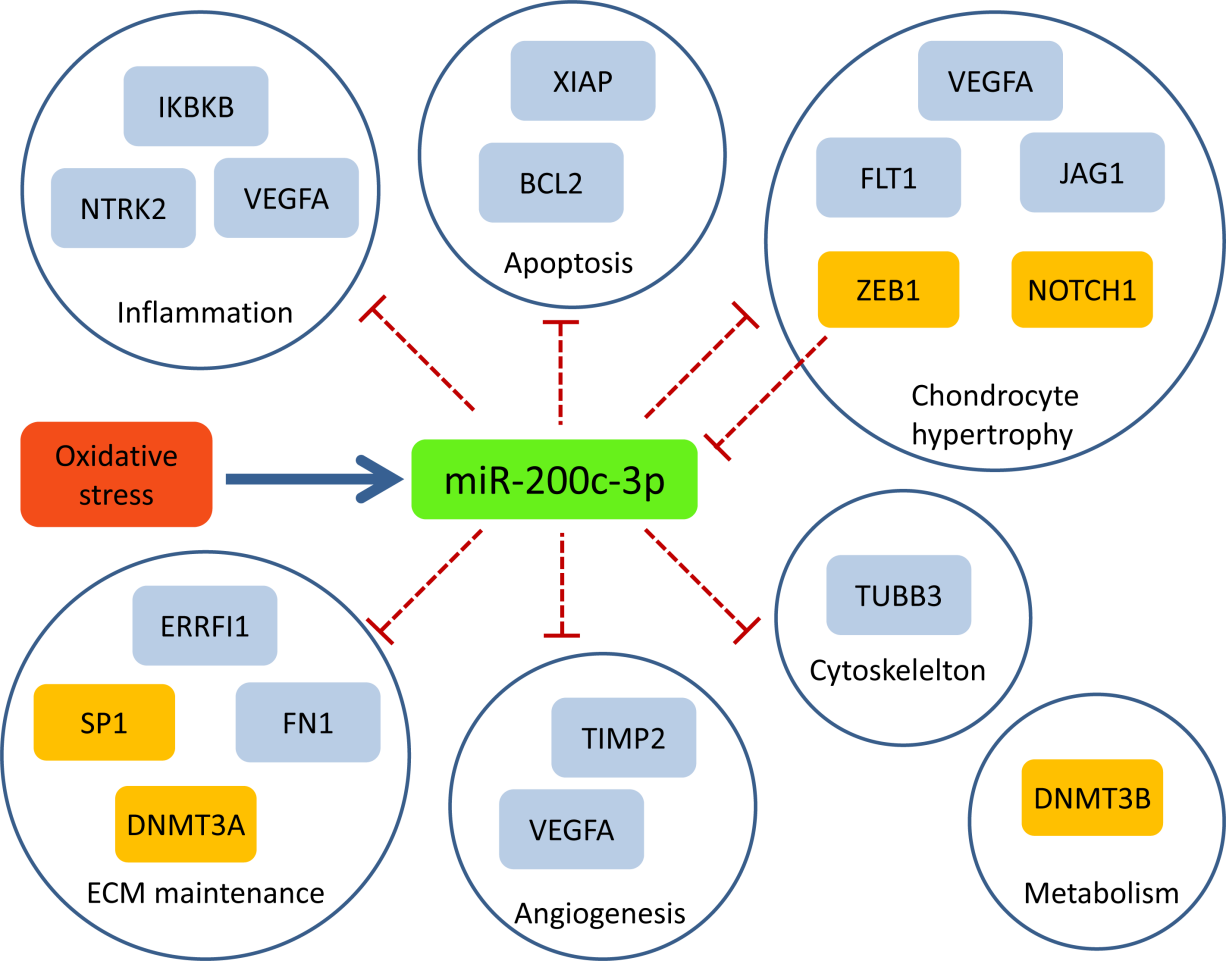
Potential role of miR-200c-3p in osteoarthritis. Oxidative stress leads to up-regulation of miR-200c-3p. Validated targets of miR-200c-3p that are involved in processes relevant to OA are shown. OA targets from targetvaliation.org are shown in blue rectangles; targets in orange rectangles have strong evidence in miRTarBase and a literature search reveals they are potential targets for OA. ZEB1 inhibits miR-200c-3p to provide a double-negative feedback loop. Red dashed lines indicate inhibition.

miR-200c-3p has the highest number of OA-related targets (S2 file). We examined the potential role of this miRNA in OA by using information from miRTarBase, and carrying out a literature search on the targets that had strong evidence (S2 Table). These targets were involved in a variety of processes that are relevant to OA including apoptosis (*BCL2*, *XIAP*), up-regulation of MMPs (*VEGFA*), chondrocyte hypertrophy (*FLT1*, *JAG1*, *VEGFA*), maintenance of ECM (*ERRFI1*, *FN1*), inflammation (*IKBKB*, *NTRK2*, *VEGFA*), angiogenesis (*TIMP2*, *VEGFA*), and maintenance of the cytoskeleton (*TUBB3*) (Fig 10). In addition, we considered 13 additional targets of miR-200c-3p that have strong validation evidence with important roles in cancer, but were not found in Open Targets as important in OA. Of these, we discovered that five targets (*DNMT3A*, *DNMT3B*, *NOTCH1*, *SP1*, and *ZEB1*) have been shown to be potentially important in cartilage maintenance, chondocyte hypertrophy, and the onset of OA (Fig 10). Interestingly, our literature search revealed that miR-200c-3p is up-regulated by oxidative stress in endothelial cells [45]. In addition, miR-200c-3p and *ZEB1* form a double-negative feedback loop that may contribute to the switch in the epithelial to mesenchymal transition in the development of cancer [46]. ZEB1 has low expression in proliferating chondrocytes but is highly expressed in the growth plate [47], where it has been shown to inhibit *IHH* [48]. This suggests the possibility that the interation between miR-200c-3p and ZEB1 may contribute to chondrocyte hypertrophy, whereby chondrocytes switch from a resting to a proliferative state, followed by differentiation.

#### miR-100-5p

miR-100-5p has six targets that are relevant for OA (S3 Table). The most relevant target is the collagenase, *MMP13*. The other targets are *PLK1*, a kinase that is involved in inducing chondrocyte apoptosis, and *FGFR3*, *FLT1*, *ID1*, *IGF1R*, genes that may all play a role in chondroctye hypertrophy.

#### miR-1826

miR-1826 has only three targets in miRTarBase but all these had strong validation evidence and were relevant for OA (S4 Table), namely *CTNNB1*, *MAP2K1*, and *VEGFC*. *CTNNB1* encodes the protein β-catenin, a key component of Wnt signalling, *MAP2K1* encodes a kinase involved in MAPK/ERK signalling, and *VEGFC* encodes a VEGF receptor.

## Discussion

We used computational modelling to explore the effect of miRNAs in different network motifs. Each model represented a biological network consisting of key components such as transcription factors, miRNAs, and target genes and a set of reactions to describe how the components interact. The generic models showed how different motifs lead to different behaviours such as bistability (positive feedback), oscillatory behaviour (negative feedback), longer lasting responses (coherent feedforward), and pulse-like responses (incoherent feedforward). We then identified some of these motifs in pathways involving miR-140 in the context of OA. Several studies have reported the effect of miR-140 on a number of different targets that are important in OA [10–13]. The aim of this study was to demonstrate how models can be built based on bioinformatics analysis and experimental data and then to show how different findings can be integrated. We constructed five different models and identified five different network motifs: positive feedback (miR-140/*SMAD3*); coherent feedforward (IL1/miR-140/*ADAMTS5*); and three types of incoherent feedforward motifs (SOX9/miR-140/RUNX2), (IL1/miR-140/*MMP13*), and (TNF-α/miR-140/*IGFBP5*). We showed that miR-140 has both positive and negative effects on cartilage. On the one hand, it inhibits translation of two major cartilage degrading enzymes, MMP-13 and ADAMTS-5. On the other hand, miR-140 reduces both IGF-1 and TGF-β signalling so that potentially less cartilage components are transcribed and, in addition, increases activation of RUNX2, which leads to up-regulation of *MMP13*. To examine the overall effect of miR-140, we combined the five models, and this model suggested that the overall effect of miR-140 would be beneficial. This agrees with observations that miR-140 is down-regulated in OA [17,49]. However, there are many other components in the different pathways of the integrated model that were omitted for simplicity in this model. Moreover, at the present state of knowledge in the field, it is quite likely that there exist relevant processes regulated by miR-140 that are currently unknown. Also, the integrated model was based on published data from different sources; some more complete than others, having, for example, different time resolutions and different experimental protocols. For all these reasons further experimental studies are required to explicitly validate parts of the network before we can have complete confidence in the integrated model’s overall behaviour. As the model is encoded in SBML, it will be possible to remove or amend parts of the network if future studies show that the current data is not robust. It will also be straight forward to add in more detail of other important mechanisms, as data become available, to produce a model that can make testable predictions.

Many miRNAs, including miR-140 have been shown to be involved in chondrogenesis [9,19]. Barter *et al*. [19] identified miR-140 and miR-455 as having an important role, with the effects of miR-140 being predominantly mediated by the miR-140-5p strand. As well as confirming many previously identified targets, they identified a new target in the Wnt signalling pathway [19]. In addition to modelling the effects of miRNAs in OA, the modelling approach described in this paper is currently being used to produce a computer simulation model of the regulatory pathways involving miRNAs in chondrogenesis. This would provide a useful tool to examine different interventions, to increase the efficiency of *in vitro* chondrogenesis.

Network motifs in biological systems have been extensively studied and modelled by Alon [3,50]. He found that some motifs are particularly common in transcriptional networks, such as the incoherent type-1 feedforward loop. There are four different incoherent feedforward loops as each of the interactions between the nodes can be either activation or repression; a type-1 loop corresponds to the arrangement shown in Figure Fi of S1 File. Alon [3,50] showed that this type of motif produces pulse-like behaviour and our model of the IL1/miR-140/*MMP13* interactions also resulted in a pulse of *MMP13* in the presence of miR-140 (Fig 5C). In our model of SOX9/miR-140/RUNX2 interactions, our incoherent network is rather different from any of the networks studied by Alon [3,50] in that one of the branches is three steps long rather than two. This gives it the features of two feedforward loops (a type-2 incoherent loop, with two inhibitory reactions centred on HDAC4, and a type-3 incoherent loop, with two activating reactions centred on miR-140). The type-2 incoherent loop is responsive to its internal signal (HDAC4) and tends to act to accelerate an off signal (switching off RUNX2), which is a desirable feature in the regulation of cartilage degradation. Alon [50] found that type-3 incoherent loops are uncommon and suggested that this was due to the poor sensitivity of the network to inputs at its internal node. In our model, this internal node corresponds to miR-140, which has the potential to increase activation of RUNX2 and hence *MMP13*. It may be that this lack of sensitivity explains why in the combined model (Fig 8), the inhibitory effects of miR-140 seem to overcome its potentially activating effect through RUNX2.

Other miRNAs have also been shown to play a role in OA, including miR-9, -98, -146 and -455 (reviewed in Le e*t al*. 2013 [9]) and more recent studies have added to the ever-growing list, e.g., miR-3085 [14]. The bioinformatics analysis (S2 File) suggests that other miRNAs such as miR-200c-3p -21-5p, -29b-3p, -100-5p and 200b-3p may also be worth further investigation. Interestingly, a recent study showed that miR-200b-3p inhibits expression of *DNMT3A* resulting in lower expression of the matrix degrading enzymes *MMP1*, *MMP3*, *MMP9* and *MMP13 [51].* In addition, another recent study examined the miR-29 family (including miR-29b-3p) in cartilage and identified a complex network of interactions between miR-29 and TGF-β, NFκB, and WNT signalling [52]. Both these studies were too recent to be included in the latest version of miRTarBase, confirming the usefulness of our bioinformatics analysis for discovering new potential targets. However, even this approach has limitations due to the time-lag between the publication of new studies and the updating of the databases. This problem was demonstrated when we carried out a search of the role of miR-200c-3p (the miRNA that was most enriched for OA targets), as this revealed other targets important in OA that were not present in Open Targets. Despite this, the analysis provides a good starting point to suggest further research into miRNAs that are currently over-looked in OA research. In identifying novel OA-associated miRNAs, we adopted the approach that miRNAs associated with cartilage development or remodelling in disease would be likely to have OA-associated genes enriched among their targets. This could be criticised for a number of reasons: the effectiveness of the approach will depend on the completeness and accuracy of annotation of the targets of a miRNA and OA related genes; OA-related genes may have multiple functions, and it may be in a different biological role that a particular miRNA is regulating them; and finally a miRNA could still be important in cartilage homeostasis or the development of OA while regulating just a few crucial genes. We also stress that focussing on particular miRNAs can lead to bias, e.g., miR-140 is known to play a role in OA but this has led to many studies of variable quality that may be over-emphasising its importance over other miRNAs. Therefore, future work should focus on validating potentially novel miRNAs that have been discovered using unbiased approaches. We carried out a search in PubMed to investigate which miRNAs from our bioinformatics analysis are novel for OA. This showed that miR-200c-3p, miR-100-5p, and miR-1826 are all good candidates for future validation. Lastly it is well-known that individual miRNAs have fairly small effects on gene expression, but many genes are regulated by many different miRNAs (referred to as miRNA target hubs) and/or have multiple sites of regulation and, so, including multiple miRNAs will lead to stronger repression of targets. To understand the effects of miRNAs in such complex networks, we will require integrative models.

## Supporting information

S1 File Model details. Results and figures for generic models of feedback and feedforward regulatory circuits involving miRNAs. Tables 1–27 giving details of all the model species, reactions and parameter values for each of the models presented in the supplementary and main paper. Table 28 Database terms for all genes/proteins present in the models.
(PDF)

S2 File hsa_mir_oa_enrichment. Excel spreadsheet showing miRNAs ranked according to their enrichment of OA-related targets.
(XLSX)

S1 Table Potential novel miRNAs for OA. (PDF)

S2 Table Potential role of hsa-miR-200c-3p in OA. Details of miR-200c-3p targets that have a role in OA.
(PDF)

S3 Table Potential role of hsa-miR-100-5p in OA. Details of miR-100-5p targets that have a role in OA.
(PDF)

S4 Table Potential role of hsa-miR-1826 in OA. Details of miR-1826 targets that have a role in OA.
(PDF)

S1 Script R script to find miRNAs with OA targets. (R)
