# Supplementary material for: Computer simulation models as a tool to investigate the role of microRNAs in osteoarthritis
Source: PLoS One. 2017 Nov 2;12(11):e0187568. doi: 10.1371/journal.pone.0187568 (PMC5695613; doi:10.1371/journal.pone.0187568)
Supplement: S1 File — Results and figures for generic models of feedback and feedforward regulatory circuits involving miRNAs. Tables 1–27 giving details of all the model species, reactions and parameter values for each of the models presented in the supplementary and main paper. Table 28 Database terms for all genes/proteins present in the models. (PDF) [file pone.0187568.s001.pdf]

Model Details

Computer simulation models as a tool to investigate the role of microRNAs in osteoarthritis

Carole J. Proctor[1,2,3], Graham R. Smith[2,4]

[1]MRC-Arthritis Research UK Centre for Integrated research into Musculoskeletal Ageing
[2]Newcastle University Institute for Ageing, Newcastle University, Newcastle upon Tyne, UK
[3]Institute of Cellular Medicine, Newcastle University, Newcastle upon Tyne, UK
[4]Bioinformatics Support Unit, Faculty of Medical Sciences, Newcastle University, Newcastle Upon Tyne, UK

Model of a positive feedback loop……………………………………………………………….2

Model of a negative feedback loop…………………………………………………………4

Model of a positive coherent feedforward loop……………………….……………………5

Model of a positive incoherent feedforward loop……………………….…………………6

Tables 1-3 Details of species, reactions and events in positive feedback model…………………….8

Tables 4-6 Details of species, reactions and events in negative feedback model………………….9

Tables 7-9 Details of species, reactions and events in negative feedback with delay model…….10

Tables 10-12 Details of species, reactions and events in coherent feedforward model.....11

Tables 13-15 Details of species, reactions and events in incoherent feedforward model.................12

Tables 16-17 Details of species and reactions in miR-140/TGFβ signalling model.………………13

Tables 18-19 Details of species and reactions in miR-140/SOX9 model…..……………………14

Tables 20-21 Details of species and reactions in miR-140/IL-1/ADAMTS5 model………………15

Tables 22-23 Details of species and reactions in miR-140/IL-1/MMP13 model……..……...……...16

Tables 24-25 Details of species and reactions in miR-140/TNF-α/IGFBP5 model………………..17

Tables 26-27 Details of additional species and reactions for integrated model ……………… …19

Table 28 Database terms for all genes/proteins in models…..……………………………………20

Supplementary Text and Figures

Model of a positive feedback loop

The motif for a positive feedback loop is shown in Figure Ai. To construct a model to illustrate this behaviour we included one microRNA (denoted by miR) and two transcription factors (TF1 and TF2) (Fig
ure Aii). The transcription of miR is inhibited by TF1 but promoted by TF2. In addition we assume that miR promotes the degradation of TF1 mRNA (TF1_mRNA) but has no effect on TF2 mRNA and so we only include transcription of TF1 in the model. As in Vera et al, 2013 [1], we assume that an external signal leads to the upregulation of a transcription factor (denoted by TF1 in our model). This reaction represents a signalling pathway which would start with a ligand binding to a receptor at the cell surface, followed by a kinase cascade and localisation of transcription factors into the nucleus, which then bind to DNA to promote transcription. The signal may be transient (as in the case when genes, which inhibit the signalling pathway, are upregulated), or persistent. TF1_mRNA is translated into protein (TF1) which we assume binds to the promoter of the miR gene to inhibit its transcription. We also assume that the degradation of TF1_mRNA is enhanced by miR. Output, from a deterministic model, shows that initially levels of miR are high and there is no production of TF1_mRNA (Figure Aiii). When the signal is turned on at time 1 hour, the level of the transcription factor (mRNA and protein) starts to increase. TF1 protein inhibits production of miR and so levels of miR decline. When the signal is switched off at time 2 hours, levels of TF1 protein continue to rise, due to pools of TF1_mRNA still being present which are now degraded more slowly due to reduced pools of miR. Eventually all the miR is depleted, so that TF1 levels stabilise but after several hours start to decline due to degradation of mRNA. Note the model includes degradation of TF1_mRNA by a miRNA independent pathway since mRNAs are targeted by multiple miRNAs. Stochastic simulations show that there is variability in the levels of miR and TF1 (Figure Aiv). Cells in which miR levels are lower have higher levels of TF1. Note that in some simulations, miR levels may start to increase again after the initial decline caused by TF1-mediated inhibition of miR synthesis. This is due to stochastic effects whereby TF2 may still bind to the miR gene even in the presence of high levels of TF1. Interestingly, if levels of TF1, TF2 and miR are low then the model predicts bi-stable behaviour with about 50% of simulated cells having high TF1 and 50% with low TF1 by 8 hours, depending on whether or not miR is totally inhibited after the signal (Figure B).

Figure A Positive feedback model. i. Network motif. **ii.** Network diagram of the model. **iii.** Output from deterministic simulation.**iv.** Output from 50 stochastic simulations. Vertical dashed green lines in **iv** indicate time that signal is turned on and off. Key for **i**: TF1=transcription factor, miR=microRNA

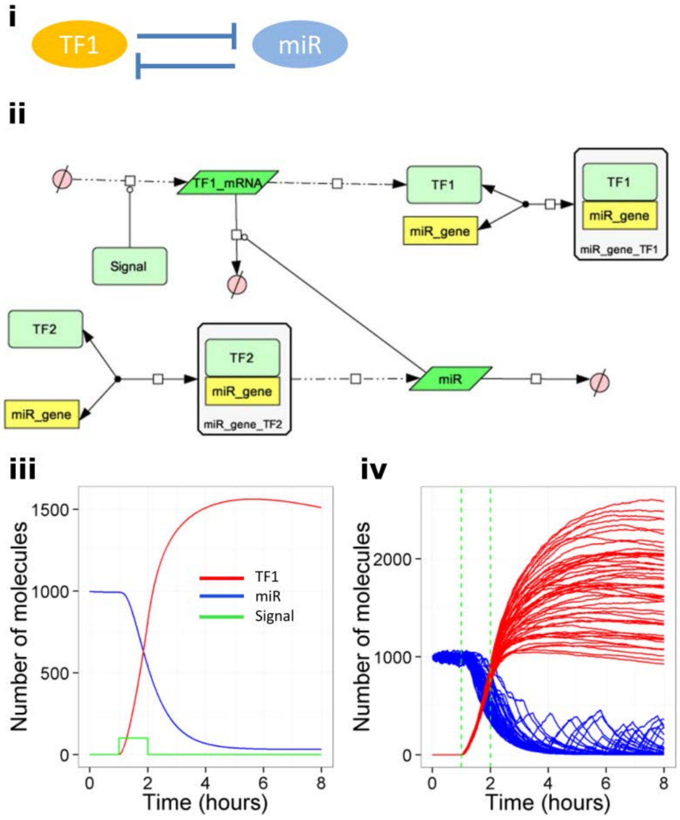

Figure B Output for positive feedback model when levels of miR and TF are low. i-viii. Eight individual stochastic simulations from 100 runs show variability of response to signal. (miR=5, TF2=10, $k_{binTF1miRgene}$=0.1, $k_{binTF2miRgene}$=0.01, k_{synmiR} =0.001, $k_{synTF1mRNA}$ =3e-5, $k_{degTF1mRNAbyMiR}$ =0.0001, k_{synTF1} =0.002,other values as in Tables S1 and S2).

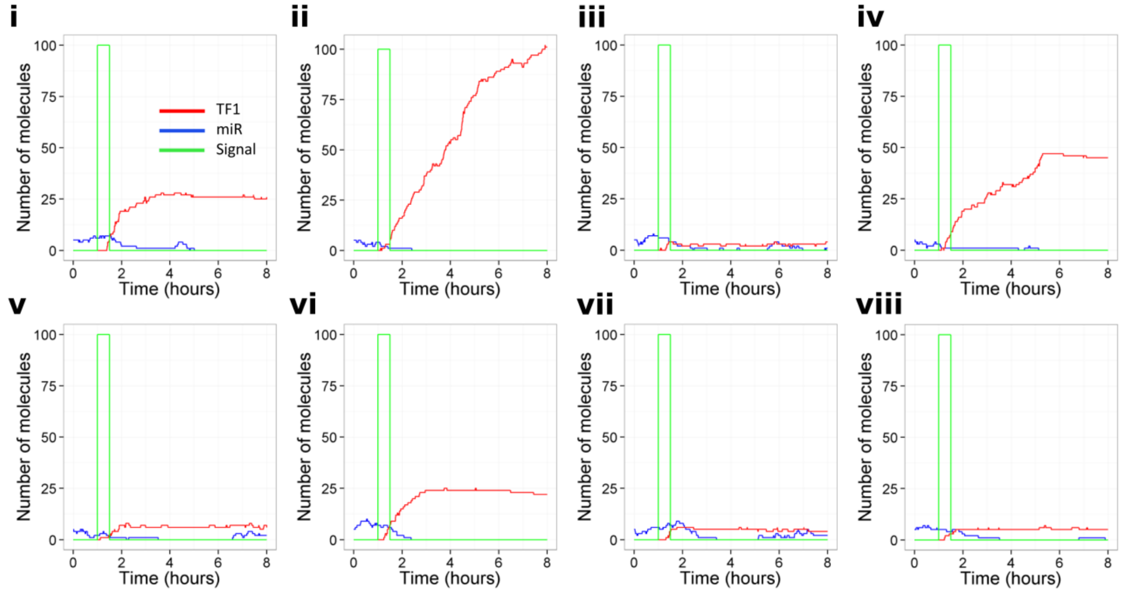

Model of a miR-regulated negative feedback loop
The motif for a negative feedback loop is shown in Figure Ci. In our model of negative regulation we included one miRNA (miR) and one transcription factor (TF1) (Figure Cii). We assumed that an external signal leads to upregulation of TF1 mRNA (TF1_mRNA). The mRNA is translated into protein which then binds to the miR promoter to activate transcription of miR. The miR enhances the degradation of TF1_mRNA and so levels of TF1 decline which then leads to less miR and so levels of TF1 are able to increase again. This cycle continues as long as the signal persists. The deterministic model output shows that a negative feedback loop leads to an initial pulse immediately after the input of the signal (at t=1 hour) of both TF1 and miR (Figure Ciii). They then decline over the next hour and reach a plateau which is above basal levels so that there is stabilisation of both miR and TF1 (Figure Ciii). Levels remain stable as long as the signal remains on. However, in reality we would expect random fluctuations in levels of TF1 and miR. The stochastic simulations capture this behaviour with levels fluctuating over time around a mean level which is above basal as long as the signal persists (Figure Civ).

Figure C Negative feedback model. i. Network motif. **ii.** Network diagram of the model. **iii.** Output from deterministic model. **iv.** Output from one stochastic simulation.

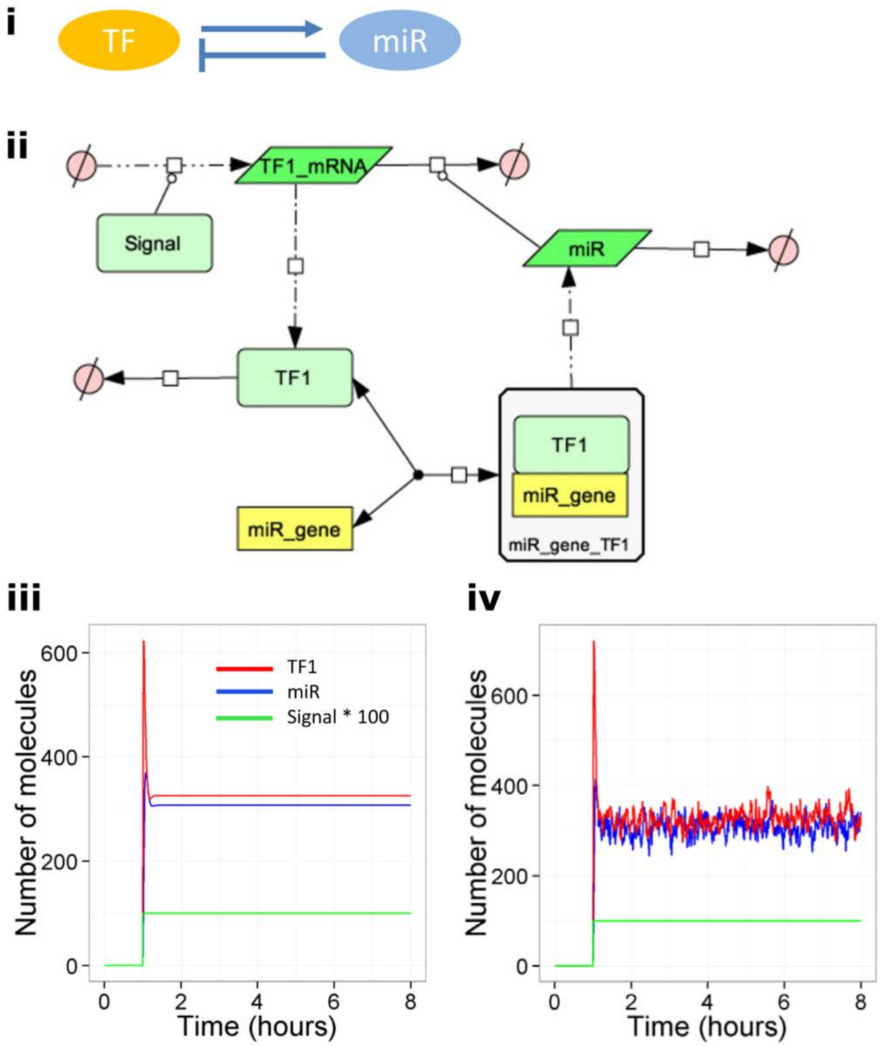

Negative feedback loops often produce regular oscillations if they also contain a delay [2] and the feedback is sufficiently strong. Therefore we modified the model in two ways. Firstly we added a delay in miR synthesis by including miR processing, and secondly we assumed that TF1 is translated in the cytoplasm and then translocates to the nucleus where it binds to the miR gene (Figure Di). This model produced regular oscillations for the stochastic model but only one peak followed by a much smaller peak in the deterministic model (Figure Dii-v). In the stochastic simulations, the first peak always occurs immediately after the signal, but further peaks occur at slightly different times (Figure Diii-v), so that these peaks will not be seen if we plot the mean level of TF1 and miR over many simulations, and would be similar to the deterministic model.

Figure D Negative feedback with delay model. i. Network diagram of model **ii.** Output from deterministic model. **iii-v.** Output from three stochastic simulations.

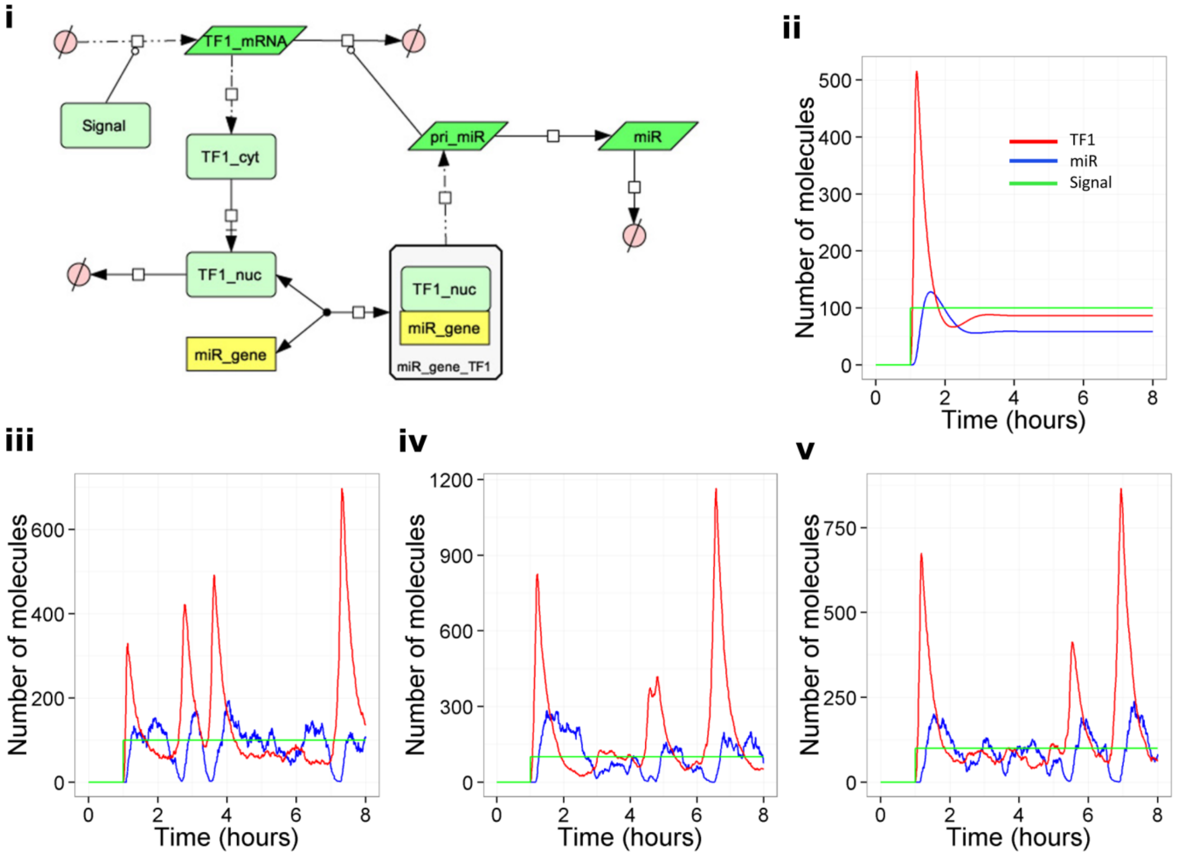

Model of a coherent feedforward loop

The motif for a coherent feedforward loop is shown in Figure Ei. Our model included one miRNA (miR), two transcription factors (TF1 and TF2) and a target gene of TF2 (TF2target) (Figure Eii). In this model, TF1 provides the signal and is switched on at time=1h and is then switched back off at time=1.5 hours and we assumed that TF1 is required for miR transcription. TF2 binds to its target gene leading to transcription. The TF2target_mRNA is degraded and we assumed that the presence of miR enhances the degradation rate (by including two degradation reactions). When TF1 is present it also binds to the TF2target_gene (competing with TF2) to inhibit transcription. So while TF1 is present, miR increases and TF2target_mRNA decreases. This is due to both less transcription and more

degradation (two inhibition loops). Both deterministic and stochastic simulation shows that the target mRNA decreases in the presence of TF1 and then increases when TF1 is switched off (Figure Eiii-iv). However, the presence of the miR negative loop not only increases the reduction of the target in the presence of the signal but also delays the time taken for the target mRNA to increase once the signal is terminated (compare Figure Eiii-iv with Figure Ev-vi in which the miR loop is removed). The additional negative feedback provided by the miR gives tighter regulation and helps to prevent leaky gene transcription, a recognised feature of coherent feedforward loops [3].

Figure E Coherent feedforward model. i. Network motif. **ii.** Network diagram of the model. **iii-iv.** miR inhibits target. **v-vi.** miR has no inhibitory effect on target. **iii**,**v.** Output from deterministic model. **iv**,**vi.** Output from one stochastic simulation.

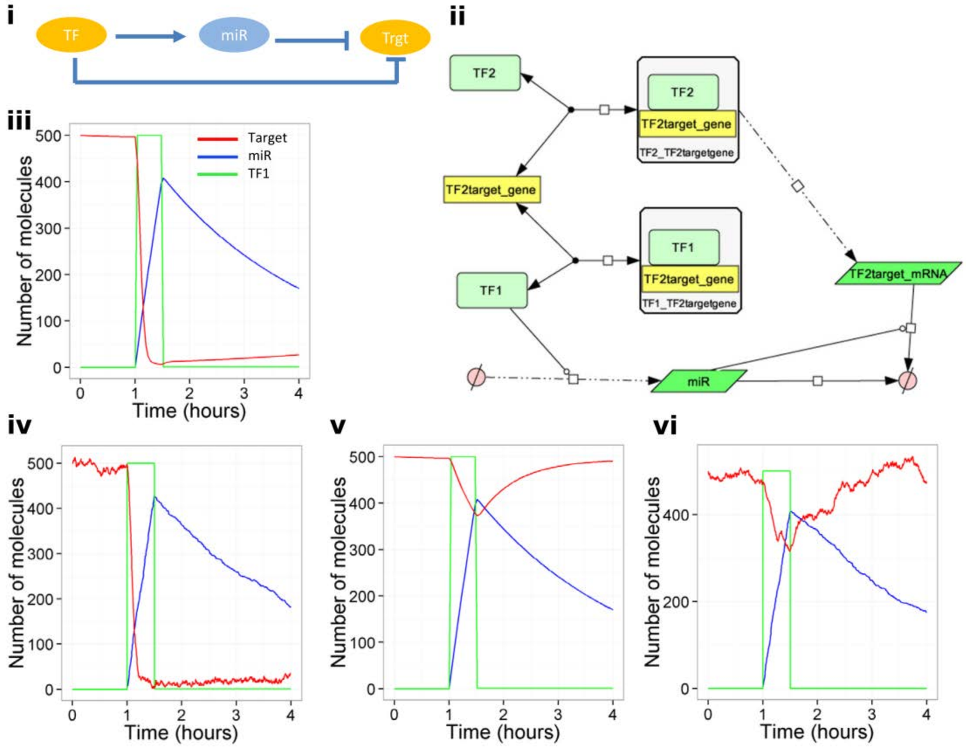

Model of an incoherent feedforward loop

The motif for an incoherent feedforward loop is shown in Figure Fi.Our model of an incoherent feedforward loop assumed that a transcription factor (TF1) upregulates both a target mRNA (Target_mRNA) and a miRNA (miR), which induces the degradation of the target mRNA (Figure Fii). During the first hour of the simulation there is no TF1, and therefore no target mRNA or miR (Figure Fiii-iv). When TF1 is switched on at 1 hour, the target mRNA rapidly increases, followed by an increase in miR which then leads to a decline in the target (Figure Fiii-iv). If inhibition of the target gene by miR is removed from the model, then the target mRNA is upregulated in the presence of TF1 and remains at high levels (Figure Fv-vi). Therefore the miRNA is acting to generate a pulse-like response of the target mRNA.

Figure F Incoherent feedforward model. i. Network motif. **ii.** Network diagram of the model. **iii-iv.** miR inhibits target. **v-vi.** miR has no inhibitory effect on target. **iii**,**v.** Output from deterministic model. **iv**,**vi.** Output from one stochastic simulation.

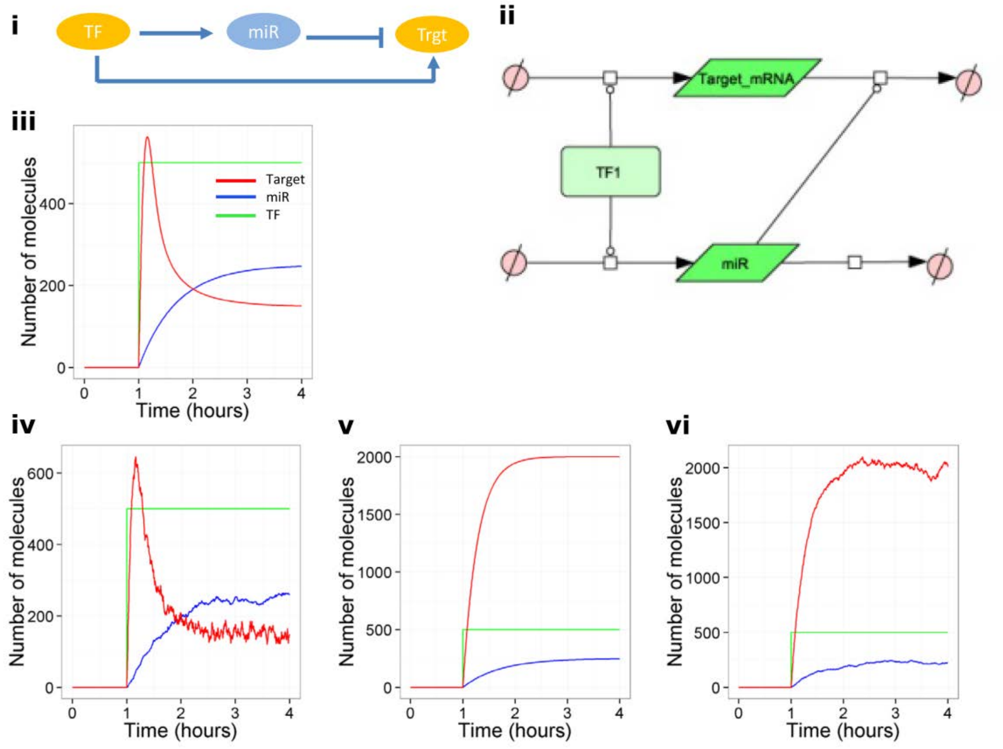

Supplementary Tables

Positive feedback model (Biomodels ID: MODEL1610100000)

Table 1 Details of species in positive feedback model

Species ID	Description	Initial Amount
miR	microRNA	1000
miR_gene	microRNA gene	2
miR_gene_TF1	miR gene bound by TF1	0
miR_gene_TF2	miR gene bound by TF2	0
Signal	Signal to activate TF1 transcription	0
TF1	Transcription factor that inhibits miR transcription	0
TF1_mRNA	Messenger RNA of TF1	0
TF2	Transcription factor that enhances miR transcription	1000

Table 2 Details of reactions in positive feedback model

Reaction ID	Reactants and products	Kinetic rate law	Parameter Value
miR_gene_TF1_binding	miR_gene + TF1 → miR_gene_TF1	$k_{binTF1miRgene}$ * miR_gene * TF1	0.002 $mol^{-1}s^{-1}$
miR_gene_TF1_release	miR_gene_TF1 → miR_gene + TF1	$k_{relTF1miRgene}$ * miR_gene_TF1	0.001 s^{-1}
miR_gene_TF2_binding	miR_gene + TF2 → miR_gene_TF2	$k_{binTF2miRgene}$ * miR_gene * TF2	0.0001 $mol^{-1}s^{-1}$
miR_gene_TF2_release	miR_gene_TF2 → miR_gene + TF2	$k_{relTF2miRgene}$ * miR_gene_TF2	0.001 s^{-1}
miR_synthesis	miR_gene_TF2 → miR_gene_TF2 + miR	k_{synMiR} * miR_gene_TF2	0.2 s^{-1}
miR_degradation	miR → Sink	k_{degMiR} * miR	0.0004 s^{-1}
TF1_transcription	Signal → Signal + TF1_mRNA	$k_{synTF1mRNA}$ * Signal	0.01 s^{-1}
TF1_mRNA_degradation	TF1_mRNA → Sink	$k_{degTF1mRNA}$ * TF1_mRNA	0.0001 s^{-1}
TF1_mRNA_degradation_via_miR	TF1_mRNA + miR → miR	$k_{degTF1mRNAbyMiR}$ * TF1_mRNA * miR	1.0e-6 $mol^{-1}s^{-1}$
TF1_translation	TF1_mRNA → TF1_mRNA + TF1	k_{synTF1} * TF1_mRNA	0.0003 s^{-1}
TF1_degradation	TF1 → Sink	k_{degTF1} * TF1	1.0e-5 s^{-1}

Table 3 Details of event in positive feedback model

Name of event	Trigger	Assignment
Activate signal	t > 3600	Signal=100
Deactivate signal	t > 7200	Signal=0

Negative feedback model (Biomodels ID: MODEL1610100001)

Table 4 Details of species in negative feedback model

Species ID	Description	Initial Amount
miR	microRNA	0
miR_gene	microRNA gene	2
miR_gene_TF1	miR gene bound by TF1	0
Signal	Signal to activate TF1 transcription	0
TF1	Transcription factor that inhibits miR transcription	0
TF1_mRNA	Messenger RNA of TF1	0

Table 5 Details of reactions in negative feedback model

Reaction ID	Reactants and products	Kinetic rate law	Parameter Value
miR_gene_TF1_binding	miR_gene + TF1 → miR_gene_TF1	$k_{binTF1miRgene}$ * miR_gene * TF1	0.005 $mol^{-1}s^{-1}$
miR_gene_TF1_release	miR_gene_TF1 → miR_gene + TF1	$k_{relTF1miRgene}$ * miR_gene_TF1	5.0 s^{-1}
miR_synthesis	miR_gene_TF1 → miR_gene_TF1 + miR	k_{synMiR} * miR_gene_TF1	5.0 s^{-1}
miR_degradation	miR → Sink	k_{degMiR} * miR	0.008 s^{-1}
TF1_transcription	Signal → Signal + TF1_mRNA	$k_{synTF1mRNA}$ * Signal	10.0 s^{-1}
TF1_mRNA_degradation	TF1_mRNA → Sink	$k_{degTF1mRNA}$ * TF1_mRNA	0.0001 s^{-1}
TF1_mRNA_degradation_via_miR	TF1_mRNA + miR → miR	$k_{degTF1mRNAbyMiR}$ * TF1_mRNA * miR	0.001 $mol^{-1}s^{-1}$
TF1_translation	TF1_mRNA → TF1_mRNA + TF1	k_{synTF1} * TF1_mRNA	0.05 s^{-1}
TF1_degradation	TF1 → Sink	k_{degTF1} * TF1	0.005 s^{-1}

Table 6 Details of event in negative feedback model

Name of event	Trigger	Assignment
Activate signal	t > 3600	Signal=1

Negative feedback with delay model (Biomodels ID: MODEL1610100002)

Table 7 Details of species in negative feedback with delay model

Species ID	Description	Initial Amount
miR	microRNA	0
miR_gene	microRNA gene	2
miR_gene_TF1	miR gene bound by TF1	0
pri_miR	Primary microRNA	0
Signal	Signal to activate TF1 transcription	0
TF1_cyt	Transcription factor that inhibits miR transcription (cytoplasmic pool)	0
TF1_nuc	Nuclear pool of TF1	0
TF1_mRNA	Messenger RNA of TF1	0

Table 8 Details of reactions in negative feedback with delay model

Reaction ID	Reactants and products	Kinetic rate law	Parameter Value
miR_gene_TF1_binding	miR_gene + TF1_nuc → miR_gene_TF1	$k_{binTF1miRgene}$ * miR_gene * TF1_nuc	0.1 $mol^{-1}s^{-1}$
miR_gene_TF1_release	miR_gene_TF1 → miR_gene + TF1_nuc	$k_{relTF1miRgene}$ * miR_gene_TF1	0.5 s^{-1}
miR_synthesis	miR_gene_TF1 → miR_gene_TF1 + pri_ miR	k_{synMiR} * miR_gene_TF1	1.0 s^{-1}
miR_processing	pri_ miR → miR	$k_{processMiR}$ * pri_miR	0.005 s^{-1}
miR_degradation	miR → Sink	k_{degMiR} * miR	0.005 s^{-1}
TF1_transcription	Signal → Signal + TF1_mRNA	$k_{synTF1mRNA}$ * Signal	0.01 s^{-1}
TF1_mRNA_degradation	TF1_mRNA → Sink	$k_{degTF1mRNA}$ * TF1_mRNA	0.0001 s^{-1}
TF1_mRNA_degradation_via_ miR	TF1_mRNA + miR → miR	$k_{degTF1mRNAbyMiR}$ * TF1_mRNA * miR	0.001 $mol^{-1}s^{-1}$
TF1_translation	TF1_mRNA → TF1_mRNA + TF1_cyt	k_{synTF1} * TF1_mRNA	0.005 s^{-1}
TF1_degradation	TF1_cyt → Sink	k_{degTF1} * TF1_cyt	0.001 s^{-1}
TF1_translocation_to_nucleus	TF1_cyt → TF1_nuc	$k_{cyt2nuc}$ * TF1_cyt	1e-5 s^{-1}
TF1_translocation_to_cytoplasm	TF1_nuc → TF1_cyt	$k_{nuc2cyt}$ * TF1_nuc	0.001 s^{-1}

Table 9 Details of event in negative feedback with delay model

Name of event	Trigger	Assignment
Activate signal	t > 3600	Signal=100

Coherent feedforward model (Biomodels ID: MODEL1610100003)

Table 10 Details of species in coherent feedforward model

Species ID	Description	Initial Amount
miR	microRNA	0
TF1	Transcription factor that activates miR transcription	0
TF1_TF2target_gene	TF1 bound to TF2 target gene to inhibit transcription	0
TF2	Transcription factor that inhibits miR transcription and activates a target (TF2_target_mRNA)	500
TF2_target_gene	Promoter of TF2 target gene	2
TF2_target_mRNA	mRNA of TF2 target	500
TF2_TF2target_gene	TF2 bound to promoter of its targt to activate transcription	0

Table 11 Details of reactions in coherent feedforward model

Reaction ID	Reactants and products	Kinetic rate law	Parameter Value
miR_synthesis	TF1 → TF1 + miR	k_{synMiR} * TF1	0.0005 s^{-1}
miR_degradation	miR → Sink	k_{degMiR} * miR	0.0001 s^{-1}
TF1_TF2target_binding	TF1 + TF2target_gene → TF1_TF2target_gene	$k_{binTF1targetgene}$ * TF1 * TF2target_gene	0.002 $mol^{-1}s^{-1}$
TF1_TF2target_release	TF1_TF2target_gene → TF1 + TF2target_gene	$k_{relTF1targetgene}$ * TF1_TF2target_gene	0.01 s^{-1}
TF2_TF2target_binding	TF2 + TF2target_gene → TF2_TF2target_gene	$k_{binTF2targetgene}$ * TF2 * TF2target_gene	0.002 $mol^{-1}s^{-1}$
TF2_TF2target_release	TF2_TF2target_gene → TF2 + TF2target_gene	$k_{relTF2targetgene}$ * TF2_TF2target_gene	0.01 s^{-1}
TF2target_gene_transcription	TF2_TF2target_gene → TF2_TF2target_gene + TF2target_mRNA	$k_{synTF2target}$ * TF2_TF2target_gene	0.1 s^{-1}
TF2target_mRNA_degradation	TF2target_mRNA → Sink	$k_{degTF2targetmRNA}$ * TF2target_mRNA	0.0004 s^{-1}
TF2target_mRNA_degradation_via_MiR	TF2target_mRNA + miR → Sink + miR	$k_{degTF2targetmRNAbyMiR}$ * TF2target_mRNA * miR	4e-5 $mol^{-1}s^{-1}$

Table 12 Details of events in coherent feedforward model

Name of event	Trigger	Assignment
Activate signal	t > 3600	TF1=500
Deactivate signal	t > 5400	TF1=0

Incoherent feedforward model (Biomodels ID: MODEL1610100004)

Table 13 Details of species in incoherent feedforward model

Species ID	Description	Initial Amount
miR	microRNA	0
TF1	Transcription factor that activates miR transcription	0
TF1target_mRNA	mRNA of TF1 target gene	0

Table 14 Details of reactions in incoherent feedforward model

Reaction ID	Reactants and products	Kinetic rate law	Parameter Value
miR_synthesis	TF1 → TF1 + miR	k_{synMiR} * TF1	$0.0002 s^{-1}$
miR_degradation	miR → Sink	k_{degMiR} * miR	$0.0004 s^{-1}$
TF1target_transcription	TF1 → TF1 + TF1target_mRNA	$k_{synTF1targetmRNA}$ * TF1	$0.004 s^{-1}$
TF1target_mRNA_degradation	TF1target_mRNA → Sink	$k_{degTF1targetmRNA}$ * TF1target_mRNA	$0.001 s^{-1}$
TF1target_mRNA_degradation_ via_MiR	TF1target_mRNA + miR → Sink + miR	$k_{degTF2targetmRNAbyMiR}$ * TF1target_mRNA * miR	5e-5 $mol^{-1}s^{-1}$

Table 15 Details of event in incoherent feedforward model

Name of event	Trigger	Assignment
Activate signal	t > 3600	TF1=500

Model of miR-140 in TGFβ signalling – a positive feedback loop (Biomodels ID: MODEL1705170000)

Table 16 Details of species in miR-140/TGFβ model

**Species ID**	**Description**	**Initial Amount**
miR140	miR-140	450
miR140_gene	miR-140 gene	2
miR140_gene_SMAD3_P	miR-140 gene bound by phospho-SMAD3	0
miR140_SMAD3_mRNA	miR-140 bound to SMAD3 mRNA	50
SMAD3	SMAD3 protein	250
SMAD3_mRNA	SMAD3 mRNA	10
SMAD3_P	Phospho-SMAD3	0
SMAD7	SMAD7 protein	0
TGFb_A	Active TGFβ	500
TGFb_I	Inactive TGFβ	0

Table 17 Details of reactions in miR-140/TGFβ model

**Reaction ID**	**Reactants and products**	**Kinetic rate law**	**Parameter Value**
SMAD3_phosphorylation	TGFb_A + SMAD3 → TGFb_A + SMAD3_P	$k_{\mathrm{phosSMAD3}}$ * TGFb_A * SMAD3	5e-5 $mol^{-1}s^{-1}$
SMAD3_dephosphorylation	SMAD3_P → SMAD3	$k_{dephosSMAD3}$ * SMAD3_P	6e-5 s^{-1}
SMAD3_transcription	Source → SMAD3_mRNA	$k_{synSMAD3mRNA}$ * Source	0.004 mol s^{-1}
SMAD3mRNA_degradation	SMAD3_mRNA → Sink	$k_{degSMAD3mRNA}$ * SMAD3_mRNA	0.0002 s^{-1}
SMAD3mRNA_degradation_by_miR140	miR140_SMAD3_mRNA → miR140	$k_{degSMAD3mRNA}$ * miR140_SMAD3_mRNA	0.0002 s^{-1}
SMAD3_translation	SMAD3_mRNA → SMAD3_mRNA + SMAD3	$k_{synSMAD3}$ * SMAD3_mRNA	0.004 s^{-1}
SMAD3_degradation	SMAD3 → Sink	$k_{degSMAD3}$ * SMAD3	0.0043 s^{-1}
SMAD3_mRNA_binding_by_miR140	SMAD3_mRNA + miR140 → miR140_SMAD3_mRNA	$k_{binSMAD3mRNAmiR140}$ * SMAD3_mRNA * miR140	0.0008 $mol^{-1}s^{-1}$
SMAD3_mRNA_miR140_release	miR140_SMAD3_mRNA → miR140 + SMAD3_mRNA	$k_{relSMAD3mRNAmiR140}$ * miR140_SMAD3_mRNA	0.001 s^{-1}
miR140_synthesis	miR140_gene → miR140_gene + miR140	$k_{synmiR140}$ * miR140_gene	0.0018 s^{-1}
miR140_degradation	miR140 → Sink	$k_{degmiR140}$ * miR140	8e-6 s^{-1}
SMAD3_miR140_gene_binding	SMAD3_P + miR140_gene → miR140_gene_SMAD3_P	$k_{binmiR140geneSMAD3}$ * SMAD3_P * miR140_gene	0.005 $mol^{-1}s^{-1}$
SMAD3_miR140_gene_release	miR140_gene_SMAD3_P → miR140_gene + SMAD3_P	$k_{relmiR140geneSMAD3}$ * miR140_gene_SMAD3_P	0.01 s^{-1}
SMAD7_snthesis	SMAD3_P → SMAD3_P + SMAD7	$k_{synSMAD7}$ * SMAD3_P	0.0005 s^{-1}
SMAD7_degradation	SMAD7 → Sink	$k_{degSMAD7}$ * SMAD7	5e-5 s^{-1}
TGFb_inactivation	TGFb_A + SMAD7 → TGFb_I	$k_{inactTGFb}$ * TGFb_A * SMAD7	0.0005 $mol^{-1}s^{-1}$

Model of miR-140 in the SOX9 pathway – an incoherent feedforward loop (Biomodels ID: MODEL1705170003)

Table 18 Details of species in miR-140/SOX9 model

**Species ID**	**Description**	**Initial Amount**
HDAC4	Histone deacetylase 4 protein	500
HDAC4_mRNA	HDAC4 mRNA	10
HDAC4_mRNA_miR140	HDAC4 mRNA bound by miR-140	0
HDAC4_RUNX2	RUNX2 protein bound by HDAC4 protein	0
HDAC4_RUNX2_gene	RUNX2 gene promoter bound by HDAC4	0
miR140	microRNA 140	10
miR140_gene	miR-140 gene	2
miR140_gene_SOX9	miR-140 gene bound by SOX9	0
MMP13_mRNA	Matrix metalloproteinase-13 mRNA	0
RUNX2	Runt related transcription factor 2 protein	500
RUNX2_gene	RUNX2 gene	2
SOX9	Transcription factor SOX9	500

Table 19 Details of reactions in miR-140/SOX9 model

**Reaction ID**	**Reactants and products**	**Kinetic rate law**	**Parameter Value**
SOX9_miR140_gene_binding	SOX9 + miR140_gene → miR140_gene_SOX9	$k_{binmiR140geneSOX9}$ * SOX9 * miR140_gene	0.002 $mol^{-1}s^{-1}$
SOX9_miR140_gene_release	miR140_gene_SOX9 → miR140_gene + SOX9	$k_{relmiR140geneSOX9}$ * miR140_gene_SOX9	0.001 s^{-1}
miR140_synthesis_by_SOX9	miR140_gene_SOX9 → miR140_gene_SOX9+ miR140	$k_{synmiR140SOX9}$ * miR140_gene_SOX9	0.0018 s^{-1}
RUNX2_inhibition_by_SOX9	RUNX2 + SOX9 → SOX9	$k_{degRUNX2}$ * RUNX2 * SOX9	1e-5 $mol^{-1}s^{-1}$
RUNX2gene_inhibition_by_ HDAC4	RUNX2_gene + HDAC4 → HDAC4_RUNX2_gene	$k_{inhibRUNX2}$ * RUNX2_gene * HDAC4	4e-6 $mol^{-1}s^{-1}$
RUNX2gene_HDAC4_release	HDAC4_RUNX2_gene → RUNX2_gene + HDAC4	$k_{relRUNX2gene}$ * HDAC4_RUNX2_gene	0.001 s^{-1}
RUNX2_synthesis	RUNX2_gene → RUNX2_gene + RUNX2	$k_{synRUNX2}$ * RUNX2_gene	0.15 s^{-1}
miR140_degradation	miR140 → Sink	$k_{degmiR140}$ * miR140	8e-6 s^{-1}
HDAC4mRNA_inhibition_by_ miR140	HDAC4_mRNA + miR140 → HDAC4_mRNA_miR140	$k_{binHDAC4mRNAmiR140}$ * HDAC4_mRNA * miR140	0.0002 $mol^{-1}s^{-1}$
HDAC4_mRNA_miR140_ Release	HDAC4_mRNA_miR140 → HDAC4_mRNA + miR140	$k_{relHDAC4mRNAmiR140}$ * HDAC4_mRNA_miR140	0.001 s^{-1}
HDAC4_transcription	Source → HDAC4_mRNA	$k_{synHDAC4mRNA}$ * Source	0.05 mol s^{-1}
HDAC4_mRNA_degradation	HDAC4_mRNA → Sink	$k_{degHDAC4mRNA}$ * HDAC4_mRNA	0.001 s^{-1}
HDAC4_mRNA_degradation_ by_miR140	HDAC4_mRNA_miR140 → miR140	$k_{degHDAC4mRNA}$ * HDAC4_mRNA_miR140	0.001 s^{-1}
HDAC4_translation	HDAC4_mRNA → HDAC4 + HDAC4_mRNA	$k_{synHDAC4}$ * HDAC4_mRNA	0.05 s^{-1}
HDAC4_degradation	HDAC4 → Sink	$k_{degHDAC4}$ * HDAC4	0.001 s^{-1}
HDAC4_RUNX2_binding	HDAC4 + RUNX2 → HDAC4_RUNX2	$k_{binHDAC4RUNX2}$ * HDAC4 * RUNX2	4e-5 $mol^{-1}s^{-1}$
HDAC4_RUNX2_release	HDAC4_RUNX2 → HDAC4 + RUNX2	$k_{relHDAC4RUNX2}$ * HDAC4_RUNX2	0.005 s^{-1}
MMP13_mRNA_synthesis_by_ RUNX2	RUNX2 → RUNX2 + MMP13_mRNA	$k_{synMMP13mRNAbyRUNX2}$ * RUNX2	4e-6 s^{-1}
MMP13_mRNA_degradation	MMP13_mRNA → Sink	$k_{degMMP13mRNA}$ * MMP13_mRNA	6.4e-6 s^{-1}

Model of miR-140 in the IL-1/ADAMTS5 pathway – a coherent feedforward loop
(Biomodels ID: MODEL1705170001)

Table 20 Details of species in miR-140/IL-1 model

Species ID	Description	Initial Amount
IL1	Interleukin-1 protein	500
miR140	microRNA 140	500
miR140_gene	miR-140 gene	2
ADAMTS5_mRNA	ADAMTS5 mRNA	0
ADAMTS5_mRNA_miR140	ADAMTS5 mRNA bound by miR-140	0

Table 21 Details of reactions in miR-140/IL-1 model

Reaction ID	Reactants and products	Kinetic rate law	Parameter Value
IL1_degradation	IL1 → Sink	k_{degIL1} * IL1	0.0004 s^{-1}
miR140_synthesis	miR140_gene → miR140_gene + miR140	$k_{synmiR140}$ * miR140_gene	0.0018 s^{-1}
ADAMTS5_mRNA_synthesis	IL1 → IL1 + ADAMTS5_mRNA	$k_{synADAMTS5mRNA}$ * IL1	0.001 s^{-1}
ADAMTS5_mRNA_binding_ by_miR140	ADAMTS5_mRNA + miR140 → ADAMTS5_mRNA_miR140	$k_{binADAMTS5mRNAmiR140}$ * ADAMTS5_mRNA * miR140	5e-5 $mol^{-1}s^{-1}$
ADAMTS5_mRNA_miR140_ release	ADAMTS5_mRNA_miR140 → ADAMTS5_mRNA + miR140	$k_{relADAMTS5mRNAmiR140}$ * ADAMTS5_mRNA_miR140	0.01 s^{-1}
ADAMTS5_mRNA_ degradation	ADAMTS5_mRNA_miR140 → miR140	$k_{degADAMTS5mRNA}$ * ADAMTS5_mRNA_miR140	0.00014 s^{-1}
miR140_degradation	miR140 → Sink	$k_{degmiR140}$ * miR140	8e-6 s^{-1}
miR140_degradation_via_IL1	miR140 + IL1 → IL1	$k_{degmiR140byIL}$ * miR140 * IL1	8e-7 s^{-1}

Model of the miR-140/IL-1/MMP-13 pathway – an incoherent feedback loop (Biomodels ID: MODEL1705170002)

Table 22 Details of species in miR-140/IL-1/MMP-13 model

Species ID	Description	Initial Amount
IL1	Interleukin-1 protein	500
miR140	microRNA 140	10
miR140_gene	miR-140 gene	2
miR140_gene_NFkB	miR-140 gene bound by NFκB	0
MMP13_mRNA	MMP-13 mRNA	25
MMP13_mRNA _miR140	MMP13_mRNA mRNA bound by miR-140	0
NFkB	Transcription factor NFκB protein	10

Table 23 Details of reactions in miR-140/IL-1/MMP-13 model

Reaction ID	Reactants and products	Kinetic rate law	Parameter Value
IL1_degradation	IL1 → Sink	k_{degIL1} * IL1	0.0004 s^{-1}
NFkB_activation	IL1 → IL1 + NFkB	*kactNFkB* * IL1	0.0005 s^{-1}
NFkB_inactivation	NFkB → Sink	*kinactNFkB* * NFkB	5e-6 s^{-1}
NFkB_miR140_gene_ binding	NFkB + miR140_gene → miR140_gene_NFkB	*kbinmiR140geneNFkB* * NFkB * miR140_gene	5e-5 $mol^{-1}s^{-1}$
NFkB_miR140_gene_ release	miR140_gene_NFkB → miR140_gene + NFkB	*krelmiR140geneNFkB* * miR140_gene_NFkB	0.001 s^{-1}
miR140_synthesis	miR140_gene_NFkB → miR140_gene_NFkB + miR140	*ksynmiR140NFkB* * miR140_gene_NFkB	0.0018 s^{-1}
MMP13_mRNA_synthesis	NFkB → NFkB + MMP13_mRNA	*ksynMMP13mRNA* * NFkB	1e-5 s^{-1}
MMP13_mRNA_binding_ by_miR140	MMP13_mRNA + miR140 → MMP13_mRNA_miR140	*kbinMMP13mRNAmiR140* * MMP13_mRNA* miR140	5e-5 $mol^{-1}s^{-1}$
MMP13_mRNA_miR140_ release	MMP13_mRNA_miR140 → MMP13_mRNA + miR140	*krelMMP13mRNAmiR140* * MP13_mRNA_miR140	0.01 s^{-1}
MMP13_mRNA_ degradation_by_miR140	MMP13_mRNA_miR140 → miR140	*kdegMMP13mRNAmiR140* * MP13_mRNA_miR140	8e-5 s^{-1}
miR140_degradation	miR140 → Sink	$k_{degmiR140}$ * miR140	8e-6 s^{-1}

Model of miR-140 in the IGF-1 signalling pathway – an incoherent feedback loop
(Biomodels ID: MODEL1705170004)

Table 24 Details of species in miR-140/TNF-α/IGFBP5 model

**Species ID**	**Description**	**Initial Amount**
ACAN	Aggrecan	0
AKT	AKT serine/threonine kinase	100
AKT_P	Phosphorylated AKT	0
IGF1	Insulin like growth factor-1	500
IGF1_IGFBP5	IGF1 bound by IGFBP5	0
IGFBP5	Insulin like growth factor binding protein 5	100
IGFBP5_mRNA	mRNAof IGFBP5	20
IGFBP5_mRNA_miR140	IGFBP5 mRNA bound by miR-140	0
IkB	NFκB inhibitor	0
IkB_NFkB	NFκB inhibited by being bound to IκB	500
JNK	Mitogen activated protein kinase	100
JNK_P	Phosphorylated JNK	0
miR140	microRNA 140	200
miR140_gene	miR-140 gene	2
miR140_gene_NFkB	miR-140 gene bound by NFκB	0
NFkB	Transcription factor NFκB protein	0
TNFa	Tumour necrosis factor	500

Table 25 Details of reactions in miR-140/TNF-α/IGFBP5 model

**Reaction ID**	**Reactants and products**	**Kinetic rate law**	**Parameter Value**
miR140_synthesis	miR140_gene → miR140_gene + miR140	$k_{synmiR140}$ * miR140_gene	0.0001 s^{-1}
NFkB_activation	TNFa + IkB_NFkB → TNFa + NFkB	$k_{actNFkB}$ * TNFa * IkB_NFkB	0.0001$mol^{-1}s^{-1}$
NFkB_miR140_gene_binding	NFkB + miR140_gene → miR140_gene_NFkB	$k_{binmiR140geneNFkB}$ * NFkB * miR140_gene	5e-5 $mol^{-1}s^{-1}$
NFkB_miR140_gene_release	miR140_gene_NFkB → miR140_gene + NFkB	$k_{relmiR140geneNFkB}$ * miR140_gene_NFkB	0.001 s^{-1}
miR140_synthesis_via_NFkB	miR140_gene_NFkB → miR140_gene_NFkB+miR140	$k_{synmiR140NFkB}$ * miR140_gene_NFkB	0.0015 s^{-1}
miR140_degradation	miR140 → Sink	$k_{degmiR140}$ * miR140	5e-6 s^{-1}
IGFBP5_transcription	Source → IGFBP5_mRNA	$k_{synIGFBP5mRNA}$ * Source	0.02 mol s^{-1}
IGFBP5_transcription_via_ JNK	JNK_P → IGFBP5_mRNA + JNK_P	$k_{synIGFBP5mRNAJNK}$ * JNK_P	0.0006 s^{-1}
IGFBP5_mRNA_degradation	IGFBP5_mRNA → Sink	$k_{degIGFBP5mRNA}$* IGFBP5_mRNA	1e-5 s^{-1}
IGFBP5_mRNA_miR140_bi nding	IGFBP5_mRNA + miR140 → IGFBP5_mRNA_miR140	$k_{binmiR140IGFBP5mRNA}$ * IGFBP5_mRNA * miR140	5e-5 $mol^{-1}s^{-1}$
IGFBP5_mRNA_miR140_rel ease	IGFBP5_mRNA_miR140 → IGFBP5_mRNA + miR140	$k_{relmiR140IGFBP5mRNA}$ * IGFBP5_mRNA_miR140	0.001 s^{-1}
IGFBP5_mRNA_degradation _by_miR140	IGFBP5_mRNA_miR140 → miR140	$k_{degIGFBP5mRNAmiR140}$ * IGFBP5_mRNA_miR140	0.0005 s^{-1}
IGFBP5_translation	IGFBP5_mRNA → IGFBP5_mRNA + IGFBP5	$k_{synIGFBP5}$ * IGFBP5_mRNA	8e-5 s^{-1}
IGFBP5_degradation	IGFBP5 → Sink	$k_{degIGFBP5}$ * IGFBP5	5e-5 s^{-1}
IGF1_IGFBP5_binding	IGF1 + IGFBP5 →IGF1_IGFBP5	$k_{binIGF1IGFBP5}$ * IGF1 * IGFBP5	5e-6 $mol^{-1}s^{-1}$
IGF1_IGFBP5_release	IGF1_IGFBP5 →IGF1 + IGFBP5	$k_{relIGF1IGFBP5}$ * IGF1_IGFBP5	0.001 s^{-1}
AKT_phosphorylation	IGF1_IGFBP5 + AKT → IGF1_IGFBP5 + AKT_P	$k_{phosAKT}$ * IGF1_IGFBP5 * AKT	0.0005 $mol^{-1}s^{-1}$
AKT_dephosphorylation	AKT_P → AKT	$k_{dephosAKT}$ * AKT_P	0.01 s^{-1}
ACAN_transcription	AKT_P → AKT_P + ACAN	$k_{synACAN}$ * AKT_P	1e-7 s^{-1}
JNK_phosphorylation	TNFa + JNK → TNFa + JNK_P	$k_{phosJNK}$ * TNFa * JNK	0.05 $mol^{-1}s^{-1}$
JNK_dephosphorylation	JNK_P → JNK	$k_{dephosJNK}$ * JNK_P	0.05 s^{-1}
TNFa_degradation	TNFa → Sink	$k_{degTNFa}$ * TNFa	0.002 s^{-1}
TNFa_synthesis	NFkB → NFkB + TNFa	$k_{synTNFa}$ * NFkB	0.001 s^{-1}
IkB_synthesis	NFkB → NFkB + IkB	k_{synIkB} * NFkB	0.005 s^{-1}
IkB_NFkB_binding	NFkB + IkB → IkB_NFkB	$k_{binIkBNFkB}$ * IkB * NFkB	0.001 $mol^{-1}s^{-1}$

Integrated model (Biomodels ID: MODEL1705170005)

Table 26 Details of additional species in the integrated model

Species ID	Description	Initial Amount
ADAMTS5	ADAMTS5 protein	0
anti_miR140	Inhibitor of miR140	0
AggFrag	Fragment of aggrecan	0
Aggrecan	Aggrecan protein in the extracellular matrix	0
Aggrecan_Collagen2	Collagen2 bound by Aggrecan in the ECM	1000
COL2A1	mRNA of Collagen2	0
ColFrag	Fragment of collagen2	0
Collagen2	Collagen2 protein in the ECM	0
miR140_anti_miR140	miR140 bound my miR140 inhibitor	0
MMP13	MMP13 protein	0
SOX9_A	Transcriptionally Active SOX9	0

Table 27 Details of additional reactions in the integrated model

Reaction ID	Reactants and products	Kinetic rate law	Parameter Value
ACAN_synthesis_by_SMAD3	SMAD3_P → SMAD3_P + ACAN	$k_{synACANSMAD3}$ * SMAD3_P	1e-6 s^{-1}
Col2_synthesis_by_SMAD3	SMAD3_P → SMAD3_P+COL2A1	$k_{synCOL2A1SMAD3}$ * SMAD3_P	1e-7 s^{-1}
SOX9_activation	SMAD3_P+SOX9→SMAD3_P + SOX9_A	$k_{actSOX9}$ * SMAD3_P * SOX9	5e-6 $mol^{-1}s^{-1}$
SOX9_inactivation	SOX9_A → SOX9	$k_{inactSOX9}$ * SOX9_A	0.0015 s^{-1}
ACAN_synthesis_by_SOX9	SOX9_A → SOX9_A + ACAN	$k_{synACANSOX9}$ * SOX9_A	1e-6 s^{-1}
Col2_synthesis_by_SOX9	SOX9_A → SOX9_A + COL2A1	$k_{synCOL2A1SOX9}$ * SOX9_A	1e-7 s^{-1}
Col2_synthesis_by_IGF1	AKT_P → AKT_P + COL2A1	$k_{synCOL2A1}$ * AKT_P	1e-8 s^{-1}
COL2A1_to_ECM	COL2A1 → Collagen2	$k_{exportCOL2A1}$ * COL2A1	0.0001 s^{-1}
ACAN_to_ECM	ACAN → Aggrecan	$k_{exportACAN}$ * ACAN	0.0001 s^{-1}
Aggrecan_Collagen2_binding	Aggrecan + Collagen2 → Aggrecan_Collagen2	$k_{binAggCol2}$ * Aggrecan * Collagen2	0.001 $mol^{-1}s^{-1}$
ADAMTS5_translation	ADAMTS5_mRNA → ADAMTS5_mRNA +ADAMTS5	$k_{synADAMTS5}$ * ADAMTS5_mRNA	1e-5 s^{-1}
ADAMTS5_removal	ADAMTS5 → Sink	$k_{degADAMTS5}$ * ADAMTS5	5e-5 s^{-1}
MMP13_translation	MMP13_mRNA → MMP13_mRNA + MMP13	$k_{synMMP13}$ * MMP13_mRNA	8e-6 s^{-1}
MMP13_removal	MMP13 → Sink	$k_{degMMP13}$ * MMP13	6.4e-6 s^{-1}
Aggrecan_degradation	Aggrecan_Collagen2+ADAMTS5 → Collagen2+ADAMTS5+AggFrag	$k_{degAggrecan}$ * Aggrecan_Collagen2 * ADAMTS5	1e-9 $mol^{-1}s^{-1}$
Collagen_degradation	Collagen2+MMP13 → MMP13+ColFrag	$k_{degCollagen}$ * Collagen2 * MMP13	1e-8 $mol^{-1}s^{-1}$
Inhibit_miR140	anti_miR140 + miR140 → miR140_anti_miR140	$k_{inhibmiR140}$ * anti_miR140 * miR140	0.001 $mol^{-1}s^{-1}$

Table 28 Database terms for all genes/proteins in models

Model species	HGNC approved symbol	HGNC ID
ACAN	ACAN	HGNC:319
ADAMTS5	ADAMTS5	HGNC:221
AKT	AKT1	HGNC:391
HDAC4	HDAC4	HGNC:14063
IGF-1	IGF1	HGNC:5464
IGFBP5	IGFBP5	HGNC:5474
IkB	NFKBIA	HGNC:7797
IL1	IL1B	HGNC:5992
JNK	MAPK8	HGNC:6881
miR140	MIR140	HGNC:31527
MMP13	MMP13	HGNC:7159
NFkB	RELA	HGNC:9955
SMAD3	SMAD3	HGNC:6769
RUNX2	RUNX2	HGNC:10472
SOX9	SOX9	HGNC:11204
SMAD7	SMAD7	HGNC:6773
TGFb	TGFB1	HGNC:11766
TNFa	TNF	HGNC:11892

HGNC - Hugo Gene Nomenclature Committee

References

1. Vera J, Lai X, Schmitz U, Wolkenhauer O. MicroRNA-regulated networks: the perfect storm for classical molecular biology, the ideal scenario for systems biology. Adv Exp Med Biol 2013; 774: 55-76.
2. Bar-Or RL, Maya R, Segel LA, Alon U, Levine AJ, Oren M. Generation of oscillations by the p53-Mdm2 feedback loop: A theoretical and experimental study. Proceedings of the National Academy of Sciences of the United States of America 2000; 97: 11250-11255.
3. Mangan S, Alon U. Structure and function of the feed-forward loop network motif. Proceedings of the National Academy of Sciences 2003; 100: 11980-11985.
