# Supplementary material for: Computer simulation models as a tool to investigate the role of microRNAs in osteoarthritis
Source: PLoS One. 2017 Nov 2;12(11):e0187568. doi: 10.1371/journal.pone.0187568 (PMC5695613; doi:10.1371/journal.pone.0187568)
Supplement: S1 Table — (PDF) [file pone.0187568.s003.pdf]

Table S1 Potential novel miRNAs for OA

miRNA	200c-3p	21-5p	29b-3p	100-5p	200b-3p	155-5p	34a-5p	125-5p	223-3p	1826	124-3p	140-5p
**Number of publications related to OA**	0	4	2	0	1	11	1	2	9	0	2	27

Search carried out in PubMed (https://www.ncbi.nlm.nih.gov/pubmed/) on 11/07/2017 with search terms ((miR-Xy-Zp OR miRXy) AND osteoarthritis)
