# Supplementary material for: Computer simulation models as a tool to investigate the role of microRNAs in osteoarthritis
Source: PLoS One. 2017 Nov 2;12(11):e0187568. doi: 10.1371/journal.pone.0187568 (PMC5695613; doi:10.1371/journal.pone.0187568)
Supplement: S2 Table — Details of miR-200c-3p targets that have a role in OA. (PDF) [file pone.0187568.s004.pdf]

Table S2 Potential role of hsa-miR-200c-3p in OA

Gene name[a]	miRTarBase ID	Evidence[b]	Publications in miRTarBase	Disease	Function/process	Relevance for OA
BCL2	MIRT006673	3 + 0	1	Lung cancer	Inhibits apoptosis.	Levels decline in OA[1]; may lead to increase in chondrocyte apoptosis.
ERRFI1	MIRT006520	1 + 1	1	Bladder cancer	Negative regulator of EGFR signalling. Regulates EMT in cancer.	ERRFI1 has been shown to be required for joint homeostasis in mice[2,3]. Up-regulated in hip OA in dogs[4]. Balance in EGFR signalling may be disturbed in OA.
FLT1	MIRT006885	3 + 0	2	Endometrial and colorectal cancer	VEGF receptor. Involved in EMT in cancer.	VEGF signalling is increased in OA and may contribute to chondrocyte hypertrophy (reviewed in[5]).
FN1	MIRT003959	3 + 2	2	Ovarian, breast and endometrial cancer	Fibronectin 1. Involved in cell motility and anoikis in cancer.	Increased in OA and highly connected to genes involved in ECM maintenance[6].
IKBKB	MIRT006884	2 + 0	2	Endometrial cancer and leiomyomas	Activates NF-κB signalling. Role in cancer is complex.	Inappropriate NF-κB signalling may play a role in OA (reviewed in[7]).
JAG1	MIRT005457	1 + 1	1	Pancreatic and breast cancer	Ligand for Notch receptors. Increased Notch signalling observed in some cancers.	Upregulated in OA[8,9]. Notch signalling in chondrocytes affects endochondral ossification and OA development[9].
NTRK2	MIRT006434	3 + 0	2	Breast cancer	Kinase, involved in BDNF signalling. May be increased in cancer leading to chemo-resistance.	Mutations in NTRK2 linked to obesity (a risk factor for OA). Increased BDNF signalling leads to inflammation in OA[10] .
TIMP2	MIRT006769	1 + 0	1	Leiomyomas	Bi-phasic regulation of MMP-2.	TIMP2 deficient mice have accelerated OA after DMM surgery due to increase in angiogenesis[11].
TUBB3	MIRT000196	3 + 2	4	Ovarian, breast and endometrial cancer	Class III member of beta-tubulin, component of microtubules. Associated with drug resistance and poor prognosis in ovarian cancer.	Colchicine (drug that inhibits tubulin and used to treat gout) is currently undergoing clinical trial in OA[12].

VEGFA	MIRT006771	2 + 0	2	Endometrial cancer and leiomyomas	Ligand that binds to FLT1 to activate VEGF signalling.	Leads to upregulation of MMPs, pro-inflammatory factors, and angiogenesis (reviewed in[5]).
XIAP	MIRT006672	3 + 0	1	Gastric and lung cancer	Inhibits apoptosis; resistance to anti-cancer drugs.	Upregulated in OA[13] ADAM15 protected chondrocytes from apoptosis via up-regulation of XIAP[13].
DNMT3A[c]	MIRT053571	3 + 0	1	Gastric cancer	Methyltransferase , mainly in *de novo* methylation during development.	Elevated in OA cartilage tissue[14]. Study showed that miR-200b-3p targets DNMT3A and that DNMT3A overexpression leads to increase in MMPs and decrease in Collagen II[14].
DNMT3B[c]	MIRT053572	3 + 0	1	Gastric cancer	Methyltransferase, mainly in *de novo* methylation during development.	Deletion of DNMT3B in chondrocytes of mice affected metabolic processes and led to early-onset OA-like pathology[15]
NOTCH1[c]	MIRT438061	3 + 0	1	Head and neck paragangliomas	Notch receptor.	Notch signalling in chondrocytes affects endochondral ossification and OA development[9]. Overexpression of Notch1 in mice to induce either sustained or transient Notch signalling resulted in early OA-like pathology or maintenance of cartilage respectively[16].
SP1[c]	MIRT053573	3 + 0	1	Gastric cancer	Transcription factor which can either repress or activate genes.	Represses transcription of the MMP inhibitor, TIMP1[17].
ZEB1[c]	MIRT002286	3 + 3	31	Ovarian, breast and other cancers	Transcription factor (repressor). Forms a double negative feedback loop with miR-200c-3p to provide switch in EMT.	Low expression in proliferating chondrocytes but highly expressed in growth plate, meniscal and articular cartilage[18]. Negatively regulated Col2A1 in chondrocytes[19]. Negative regulator of Ihh in the growth plate[20].

Notes: [a]HGNC (HUGO Gene Nomenclature Committee) approved symbol. [b] Strength of evidence is given by the number of different validation methods. The first integer represents strong evidence (reporter assay, Western blot or qPCR). The second integer represents less strong evidence (microarray, NGS, pSILAC or other). [c]Genes which are strongly validated targets of miR-200c-3p but are not included in validationtargets.org as OA targets.

Abbreviations: DMM - destabilization of the medial meniscus; ECM – extracellular matrix; EMT - epithelial to mesenchymal transition

References

1. Karaliotas, G. I., Mavridis, K., Scorilas, A. & Babis, G. C. Quantitative analysis of the mRNA expression levels of BCL2 and BAX genes in human osteoarthritis and normal articular cartilage: An investigation into their differential expression. *Molecular medicine reports* **12**, 4514-4521, doi:10.3892/mmr.2015.3939 (2015).
2. Pest, M. A., Russell, B. A., Zhang, Y. W., Jeong, J. W. & Beier, F. Disturbed cartilage and joint homeostasis resulting from a loss of mitogen-inducible gene 6 in a mouse model of joint dysfunction. *Arthritis Rheumatol* **66**, 2816-2827, doi:10.1002/art.38758 (2014).
3. Staal, B., Williams, B. O., Beier, F., Vande Woude, G. F. & Zhang, Y. W. Cartilage-specific deletion of Mig-6 results in osteoarthritis-like disorder with excessive articular chondrocyte proliferation. *Proceedings of the National Academy of Sciences of the United States of America* **111**, 2590-2595, doi:10.1073/pnas.1400744111 (2014).
4. Mateescu, R. G., Todhunter, R. J., Lust, G. & Burton-Wurster, N. Increased MIG-6 mRNA transcripts in osteoarthritic cartilage. *Biochem Biophys Res Commun* **332**, 482-486, doi:10.1016/j.bbrc.2005.04.144 (2005).
5. Murata, M., Yudoh, K. & Masuko, K. The potential role of vascular endothelial growth factor (VEGF) in cartilage. *Osteoarthritis and Cartilage* **16**, 279-286, doi:http://dx.doi.org/10.1016/j.joca.2007.09.003 (2008).
6. Ramos, Y. F. M. *et al.* Genes involved in the osteoarthritis process identified through genome wide expression analysis in articular cartilage; the RAAK study. *PLoS ONE* **9**, e103056, doi:10.1371/journal.pone.0103056 (2014).
7. Rigoglou, S. & Papavassiliou, A. G. The NF-κB signalling pathway in osteoarthritis. *The International Journal of Biochemistry & Cell Biology* **45**, 2580-2584, doi:http://dx.doi.org/10.1016/j.biocel.2013.08.018 (2013).
8. Karlsson, C., Brantsing, C., Egell, S. & Lindahl, A. Notch1, Jagged1, and HES5 are abundantly expressed in osteoarthritis. *Cells Tissues Organs* **188**, 287-298, doi:10.1159/000121610 (2008).
9. Hosaka, Y. *et al.* Notch signaling in chondrocytes modulates endochondral ossification and osteoarthritis development. *Proceedings of the National Academy of Sciences of the United States of America* **110**, 1875-1880, doi:10.1073/pnas.1207458110 (2013).
10. Simão, A. P. *et al.* Involvement of BDNF in knee osteoarthritis: the relationship with inflammation and clinical parameters. *Rheumatology International* **34**, 1153-1157, doi:10.1007/s00296-013-2943-5 (2014).
11. Mi, M. *et al.* TIMP2 deficient mice develop accelerated osteoarthritis via promotion of angiogenesis upon destabilization of the medial meniscus. *Biochem Biophys Res Commun* **423**, 366-372, doi:10.1016/j.bbrc.2012.05.132 (2012).
12. Leung, Y.-Y. *et al.* Colchicine effectiveness in symptom and inflammation modification in knee osteoarthritis (COLKOA): study protocol for a randomized controlled trial. *Trials* **16**, 200, doi:10.1186/s13063-015-0726-x (2015).
13. Böhm, B. *et al.* ADAM15 exerts an antiapoptotic effect on osteoarthritic chondrocytes via up-regulation of the X-linked inhibitor of apoptosis. *Arthritis Rheum.* **62**, 1372-1382, doi:10.1002/art.27387 (2010).
14. Wu, J. *et al.* Effect of the interaction between MiR-200b-3p and DNMT3A on cartilage cells of osteoarthritis patients. *J. Cell. Mol. Med.*, n/a-n/a, doi:10.1111/jcmm.13152 (2017).
15. Shen, J. *et al.* DNA methyltransferase 3b regulates articular cartilage homeostasis by altering metabolism. *JCI Insight* **2**, e93612, doi:10.1172/jci.insight.93612 (2017).

16 Liu, Z. *et al.* A dual role for NOTCH signaling in joint cartilage maintenance and osteoarthritis. *Science Signaling* **8**, ra71-ra71, doi:10.1126/scisignal.aaa3792 (2015).
17 Dean, G., Young, D. A., Edwards, D. R. & Clark, I. M. The human tissue inhibitor of metalloproteinases (TIMP)-1 gene contains repressive elements within the promoter and intron 1. *J Biol Chem* **275**, 32664-32671 (2000).
18 Davies, S. R., Sakano, S., Zhu, Y. & Sandell, L. J. Distribution of the transcription factors Sox9, AP-2, and [delta]EF1 in adult murine articular and meniscal cartilage and growth plate. *J. Histochem. Cytochem.* **50**, 1059-1065, doi:doi:10.1177/002215540205000808 (2002).
19 Murray, D., Precht, P., Balakir, R. & Horton, W. E., Jr. The transcription factor deltaEF1 is inversely expressed with type II collagen mRNA and can repress Col2a1 promoter activity in transfected chondrocytes. *J. Biol. Chem.* **275**, 3610-3618 (2000).
20 Bellon, E., Luyten, F. P. & Tylzanowski, P. δ-EF1 is a negative regulator of *Ihh* in the developing growth plate. *The Journal of cell biology* **187**, 685-699, doi:10.1083/jcb.200904034 (2009).
