# Supplementary material for: Computer simulation models as a tool to investigate the role of microRNAs in osteoarthritis
Source: PLoS One. 2017 Nov 2;12(11):e0187568. doi: 10.1371/journal.pone.0187568 (PMC5695613; doi:10.1371/journal.pone.0187568)
Supplement: S3 Table — Details of miR-100-5p targets that have a role in OA. (PDF) [file pone.0187568.s005.pdf]

Table S3 Potential role of hsa-miR-100-5p in OA

Gene name[a]	miRTarBase ID	Evidence[b]	Publications in miRTarBase	Disease	Function/process	Relevance for OA
FGFR3	MIRT003419	4 + 1	4	Oral, bladder, and prostate cancer	Fibroblast growth factor receptor. Regulation of differentiation, cell proliferation and apoptosis.	FGFR3 may have an anabolic role in cartilage (reviewed in[1]). FGFR3 decreases knee OA progression in mice by inhibiting IHH signalling[2].
FLT1	MIRT006850	1 + 0	1	(Study in COS-7 cells undergoing angiogenesis)	VEGF receptor. Involved in EMT and cancer.	VEGF signalling is increased in OA and may contribute to chondrocyte hypertrophy (reviewed in[3]).
ID1	MIRT003418	2 + 1	1	Oral cancer	Transcriptional regulator. Negatively regulates bHLH TFs. Involved in many cellular processes.	May have a role in proliferating chondrocytes[4]. Up-regulated by catabolic TGF-β/ALK1 pathway[5].
IGF1R	MIRT006429	3 + 1	4	Leukaemia, bone, and adrenocortical cancer. Type 2 diabetes	Insulin-like growth factor 1 receptor. Anti-apoptotic. Activates JAK/STAT pathway.	Regulates osterix expression and cartilage mineralisation during endochondral ossification[6]. Being studied in context of tissue engineering[7].
MMP13	MIRT003420	2 + 2	1	Oral cancer	Collagenase that degrades Collagen II.	Expressed in OA cartilage and is most potent collagenase that degrades Collagen II[8].
PLK1	MIRT000382	3 + 1	4	Cervical, liver, lung and throat cancer	Kinase that is highly expressed in mitosis.	Involved in chondrocyte apoptosis[9].

Notes: [a]HGNC (HUGO Gene Nomenclature Committee) approved symbol. [b]Strength of evidence is given by the number of different validation methods. The first integer represents strong evidence (reporter assay, Western blot or qPCR). The second integer represents less strong evidence (microarray, NGS, pSILAC or other).

References

1 Ellman, M. B. *et al.* Fibroblast growth factor control of cartilage homeostasis. *J. Cell. Biochem.* **114**, 735-742, doi:10.1002/jcb.24418 (2013).

2 Tang, J. *et al.* Fibroblast growth factor receptor 3 inhibits osteoarthritis progression in the knee joints of adult mice. *Arthritis & Rheumatology* **68**, 2432-2443, doi:10.1002/art.39739 (2016).

3 Murata, M., Yudoh, K. & Masuko, K. The potential role of vascular endothelial growth factor (VEGF) in cartilage. *Osteoarthritis and Cartilage* **16**, 279-286, doi:http://dx.doi.org/10.1016/j.joca.2007.09.003 (2008).

4 Asp, J., Thornemo, M., Inerot, S. & Lindahl, A. The helix-loop-helix transcription factors Id1 and Id3 have a functional role in control of cell division in human normal and neoplastic chondrocytes. *FEBS Lett.* **438**, 85-90, doi:10.1016/S0014-5793(98)01268-X (1998).

5 Blaney Davidson, E. N. *et al.* Increase in ALK1/ALK5 ratio as a cause for elevated MMP-13 expression in osteoarthritis in humans and mice. *J Immunol* **182**, 7937-7945, doi:10.4049/jimmunol.0803991 (2009).

6 Heilig, J., Paulsson, M. & Zaucke, F. Insulin-like growth factor 1 receptor (IGF1R) signaling regulates osterix expression and cartilage matrix mineralization during endochondral ossification. *Bone* **83**, 48-57, doi:http://dx.doi.org/10.1016/j.bone.2015.10.007 (2016).

7 Witt, A. *et al.* Gene expression analysis of growth factor receptors in human chondrocytes in monolayer and 3D pellet cultures. *Int J Mol Med* **40**, 10-20, doi:10.3892/ijmm.2017.2994 (2017).

8 Mitchell, P. G. *et al.* Cloning, expression, and type II collagenolytic activity of matrix metalloproteinase-13 from human osteoarthritic cartilage. *The Journal of Clinical Investigation* **97**, 761-768, doi:10.1172/JCI118475 (1996).

9 Zhu, F., Wang, P., Kontrogianni-Konstantopoulos, A. & Konstantopoulos, K. Prostaglandin (PG)D2 and 15-deoxy-[Delta]12,14-PGJ2, but not PGE2, mediate shear-induced chondrocyte apoptosis via protein kinase A-dependent regulation of polo-like kinases. *Cell Death Differ* **17**, 1325-1334, doi:http://www.nature.com/cdd/journal/v17/n8/suppinfo/cdd201013s1.html (2010).
