# Supplementary material for: Computer simulation models as a tool to investigate the role of microRNAs in osteoarthritis
Source: PLoS One. 2017 Nov 2;12(11):e0187568. doi: 10.1371/journal.pone.0187568 (PMC5695613; doi:10.1371/journal.pone.0187568)
Supplement: S4 Table — Details of miR-1826 targets that have a role in OA. (PDF) [file pone.0187568.s006.pdf]

Table S4 Potential role of hsa-miR-1826 in OA

Gene name[a]	miRTarBase ID	Evidence[b]	Publications in miRTarBase	Disease	Function/process	Relevance for OA
CTNNB1	MIRT006602	2 + 0	2	Bladder and renal cancer	Also known as β-catenin. Key co-activator for transcription factors in canonical Wnt signalling.	Aberrant Wnt signalling may contribute to OA but role is complex (reviewed in[1,2]).
MAPK21	MIRT006601	2 + 0	2	Bladder and renal cancer	Kinase involved in MAPK/ERK signalling.	MAPK/ERK signalling may regulate expression of pro-inflammatory cytokines and MMPs[3]
VEGFC	MIRT006603	2 + 0	1	Bladder cancer	Growth factor that binds to receptors to activate VEGF signalling.	VEGF signalling may play a role in OA[4].

Notes: [a]HGNC (HUGO Gene Nomenclature Committee) approved symbol. [b] Strength of evidence is given by the number of different validation methods. The first integer represents strong evidence (reporter assay, Western blot or qPCR). The second integer represents less strong evidence (microarray, NGS, pSILAC or other).

References

1 Luyten, F. P., Tylzanowski, P. & Lories, R. J. Wnt signaling and osteoarthritis. *Bone* **44**, 522-527, doi:http://dx.doi.org/10.1016/j.bone.2008.12.006 (2009).

2 Sassi, N. *et al.* WNT signaling and chondrocytes: from cell fate determination to osteoarthritis physiopathology. *J. Recept. Signal Transduct.* **34**, 73-80, doi:10.3109/10799893.2013.863919 (2014).

3 Loeser, R. F., Erickson, E. A. & Long, D. L. Mitogen-activated protein kinases as therapeutic targets in osteoarthritis. *Curr. Opin. Rheumatol.* **20**, 581-586, doi:10.1097/BOR.0b013e3283090463 (2008).

4 Murata, M., Yudoh, K. & Masuko, K. The potential role of vascular endothelial growth factor (VEGF) in cartilage. *Osteoarthritis and Cartilage* **16**, 279-286, doi:http://dx.doi.org/10.1016/j.joca.2007.09.003 (2008).
